# Supplementary material for: Synthesis of 4′-Substituted Carbocyclic Uracil Derivatives and Their Monophosphate Prodrugs as Potential Antiviral Agents
Source: Viruses. 2023 Feb 16;15(2):544. doi: 10.3390/v15020544 (PMC9962574; doi:10.3390/v15020544)
Supplement: Supplementary file 1 [file viruses-15-00544-s001.zip › viruses-2212255-supplementary.pdf]

Supporting Information

1- General Notes

Anhydrous solvents were purchased from Aldrich Chemical Company, Inc. (Milwaukee, Wisconsin, USA). Reagents were purchased from commercial sources. Unless noted otherwise, the materials used in the examples were obtained from readily available commercial suppliers or synthesized by standard methods known to one skilled in the art of organic chemistry synthesis. ^{1}H and ^{13}C spectra were obtained on a Bruker Ascend™ 400 spectrometer (Bruker BioSpin Corporation, Billerica, MA, USA) at rt and reported in ppm downfield from internal tetramethylsilane (for ^{1}H-NMR). NMR processing was performed with MestReNova version 10.0.2-15465. Deuterium exchange and decoupling experiments were utilized to confirm proton assignments. Signal multiplicities are represented by s (singlet), d (doublet), dd (doublet of doublets), t (triplet), q (quadruplet), br (broad), bs (broad singlet), m (multiplet). All J-values are in Hz and calculated by Mnova or MestReNova programs (V 14.1.1). Mass spectra were determined on a Waters Acquity UPLC using electrospray ionization (Waters Corporation, Milford, MA, USA). Analytic TLC was performed on Analtech GHLF silica gel plates (Analtech, Newark, DE, USA), and preparative TLC on Analtech GF silica gel plates (Analtech, Newark, DE, USA). Column chromatography was performed on Combiflash R_f200 or via reverse-phase high performance liquid chromatography.

2- Synthesis of intermediate **20**.

2.1- Experimental procedures and data.

Procedure for the synthesis of 1-((1*R*,2*S*,3*R*,4*R*)-2,3-bis((*tert*-butyldimethylsilyl)oxy)-4-(hydroxymethyl)cyclopentyl)pyrimidine-2,4(1*H*,3*H*)-dione (16). To a stirred solution of reported nucleoside (**15**) [1,2,3]. (3 g, 12.4 mmol) and imidazole (6.34 g, 99 mmol) in DMF (62 mL) was added TBDMSCl (7.44 g, 49.5 mmol). The reaction mixture was stirred at 50 °C for 3 days. After completion of the reaction, DMF was evaporated, and the residue was partitioned between ethyl acetate (200 mL) and sat. $NaHCO_3$ (200 mL). The layers were separated, and the aqueous layer was extracted with ethyl acetate (3 x 75 mL). Organic phases were combined, washed with brine (150 mL), dried over $MgSO_4$, filtered, and concentrated *in vacuo*. The crude product was purified by flash chromatography (ethyl acetate/hexane 0/100 to 100/0) to give the corresponding *per*-silylated nucleoside (6.51 g, 90%) as a white foam. To a stirred solution of the *per*-silylated nucleoside (6.51 g, 11.1 mmol) in THF (40 mL) was added a solution of aqueous TFA (20 mL, TFA/water 1:1) at 0 °C. After stirring for 3 h at 0 °C, the reaction mixture was neutralized with sat. $NaHCO_3$ and extracted with ethyl acetate (3 x 80 mL). The organic layers were combined, washed with brine (100 mL), dried over $MgSO_4$, filtered, and concentrated *in vacuo*. The crude product was purified by flash chromatography (ethyl acetate/hexane 0/100 to 100/0) to give the title compound (**10**) (3.7 g, 69%) as a white foam. ^{1}H NMR (600 MHz, MeOD-*d₄*) δ 7.67 (d, 1H, *J* = 8.0 Hz), 5.68 (d, 1H, *J* = 7.9 Hz), 4.78 (q, 1H, *J* = 9.5 Hz), 4.43 (dd, 1H, *J* = 9.6, 4.1 Hz), 4.01 (d, 1H, *J* = 4.2 Hz), 3.59 (d, 2H, *J* = 6.3 Hz), 2.25 (dt, 1H, *J* = 13.6, 9.9 Hz), 2.17 – 2.10 (m, 1H), 1.65 (ddd, 1H, *J* = 13.7, 9.2, 4.6 Hz), 0.94 (s, 9H), 0.86 (s, 9H), 0.13 (s, 3H), 0.11 (s, 3H), 0.05 (s, 3H), -0.05 (s, 3H). δ ^{13}C NMR (151 MHz, MeOD-d_4) δ 166.3, 152.9, 146.3, 102.3, 76.3, 76.2, 64.7, 64.4, 47.2, 26.9, 26.4, 26.4, 18.9, 18.8, -3.9, -4.1, -4.18, -4.75. HRMS-ESI (*m*/*z*) $[M+H]^+$ calcd. 471.2632. for $C_{22}H_{43}N_2O_5Si_2$: found 471.2703.

Procedure for the synthesis of 1-((1*R*,2*S*,3*R*)-2,3-bis((*tert*-butyldimethylsilyl)oxy)-4,4-bis(hydroxymethyl)cyclopentyl)pyrimidine-2,4(1*H*,3*H*)-dione (17). Under argon, Dess-Martin periodinane (4 g, 9.44 mmol) was added to a solution of (**16**) (3.7 g, 7.87 mmol) in dichloromethane (60 mL) and pyridine (5.1 mL, 62.9 mmol) at 0 °C. The reaction mixture was stirred at room temperature for 16 h and then diluted with dichloromethane (50 mL) and sat. $NaHCO_3$ (40 mL). After filtration of the precipitate, the aqueous layer was extracted with dichloromethane (5 x 30 mL). The organic layers were then combined, washed with brine (100 mL), dried over $MgSO_4$, filtered, and concentrated *in vacuo*. The crude product was purified by flash chromatography (ethyl acetate/H\hexane 0/100 to 100/0). The resulting white solid was dissolved at 0 °C in 1,4-dioxane (84 mL), follow by the addition of aqueous formaldehyde solution (37%, 3.3 mL, 42.4 mmol) and NaOH solution (2 M, 8.4 mL, 16.9 mmol). The mixture was stirred for 10 min at 0 °C and then at room temperature overnight. The reaction was then cooled down to 0 °C before addition of $NaBH_4$ (1 g, 27.1 mmol). After being stirred for 1.5 h at room temperature, the mixture was quenched with sat. NH_4Cl (100 mL), extracted with ethyl acetate (5 x 40 mL). The organic layers were combined, washed with brine (100 mL), dried over $MgSO_4$, filtered, and concentrated *in*

vacuo. The crude product was purified by flash chromatography (methanol/dichloromethane 0/100 to 10/90) to give the title compound (**11**) (2.75 g, 70% over 3 steps) as a white solid. ^{1}H NMR (600 MHz, MeOD-d_4) δ 7.69 (d, 1H, *J* = 8.0 Hz), 5.69 (d, 1H, *J* = 7.9 Hz), 4.74 (dt, 1H, *J* = 10.4, 8.5 Hz), 4.66 (dd, 1H, *J* = 8.5, 4.0 Hz), 4.14 (d, 1H, *J* = 4.0 Hz), 3.74-3.62 (m, 4H), 2.06-1.99 (m, 1H), 1.69 (dd, 1H, *J* = 14.0, 8.5 Hz), 0.96 (s, 9H), 0.89 (s, 9H), 0.15 (s, 3H), 0.13 (s, 3H), 0.05 (s, 3H), -0.01 (s, 3H). ^{13}C NMR (151 MHz, MeOD-d_4) δ 166.3, 152.7, 146.7, 102.4, 77.1, 77.0, 65.9, 65.2, 64.5, 50.2, 31.2, 26.7, 26.7, 19.2, 19.0, -3.3, -3.6, -4.1, -4.2. HRMS-ESI (*m*/*z*) [M+H]$^+$ calcd. 501.2738. for $C_{23}H_{45}N_2O_6Si_2$: found 501.2811.

Procedure for the synthesis of 1-((1*R*,2*S*,3*R*,4*S*)-2,3-bis((*tert*-butyldimethylsilyl)oxy)-4-(((*tert*-butyldimethylsilyl)oxy)methyl)-4-(hydroxymethyl)cyclopentyl)pyrimidine-2,4(1*H*,3*H*)-dione (20). A mixture of **17** (2.05 g, 4.1 mmol) and dimethoxytrityl chloride (1.76 g, 4.92 mmol) in dichloromethane/pyridine (15.1 mL, 3:1) was stirred for 24 h at room temperature. The mixture was diluted with ethyl acetate (200 mL), and then washed with HCl (1M, 50 mL), sat. $NaHCO_3$ (50 mL) and sat. brine (50 mL). The organic layer was dried over $MgSO_4$, filtered, and concentrated *in vacuo*. The crude product was purified by flash chromatography (ethyl acetate/hexane 0/100 to 50/50) to give compound **18** (1.84 g, 56%) as a yellow foam. To a stirred solution of nucleoside (**18**) (1.84 g, 2.3 mmol) and imidazole (1.05 g, 16.5 mmol) in DMF (12.7 mL) was added TBDMSCl (0.83 g, 5.52 mmol). The reaction mixture was stirred at 50 °C for 24 h. After completion of the reaction, DMF was evaporated, and the residue was partitioned between ethyl acetate (100 mL) and sat. $NaHCO_3$ (100 mL). The layers were separated, and the aqueous layer was extracted with ethyl acetate (3 x 40 mL). The organic layers were combined, washed with brine (50 mL), dried over $MgSO_4$, filtered, and concentrated *in vacuo*. The crude product was purified by flash chromatography (ethyl acetate/hexane 0/100 to 50/50) to give compound (**19**) (2.0 g, 95%) as a white foam. Compound (**19**) was dissolved in a mixture of THF (5 mL) and aqueous 80% AcOH (43 mL) at 0 °C and stirred for 3 h at room temperature. The mixture was quenched with solid $NaHCO_3$ (pH = 7) diluted with ethyl acetate (200 mL). The organic layer was washed with water (100 mL) and brine (75 mL), dried over $MgSO_4$, filtered, and concentrated *in vacuo*. The crude product was purified by flash chromatography (ethyl acetate/hexane 0/100 to 50/50) to give the title compound (**20**) (0.79 g, 59%) as a white foam. ^{1}H NMR (400 MHz, Me_2CO-d_6) δ 9.95 (bs, 1H), 7.89 (d, 1H, *J* = 8.0 Hz), 5.57 (d, 1H, *J* = 7.9 Hz), 4.71-4.68 (m, 2H), 4.27 (d, 1H, J = 3.6 Hz), 3.82-3.79 (m, 2H), 3.76-3.70 (m, 2H), 3.50 (t, 1H, *J* = 5.2 Hz), 2.1-2.07 (m, 1H), 1.73-1.67 (m, 1H), 0.98 (s, 9H), 0.94 (s, 9H), 0.89 (s, 9H), 0.13 (s, 3H), 0.11 (s, 3H), 0.06 (s, 3H), 0.008 (s, 3H), -0.01 (s, 3H), -0.02 (s, 3H). ^{13}C NMR (101 MHz, Me_2CO-d_6) δ 163.5, 151.8, 145.7, 102.1, 76.1, 65.9, 64.8, 64.2, 50.3, 26.6, 26.5, 26.3, 18.9, 18.7, -3.4, -3.6, -4.1, -4.1, -5.2, -5.3. HRMS-ESI (*m*/*z*) [M+H]$^+$ calcd. 615.3603. for $C_{29}H_{59}N_2O_6Si_3$: found 615.3690.

2.2- NMR spectra **16**.

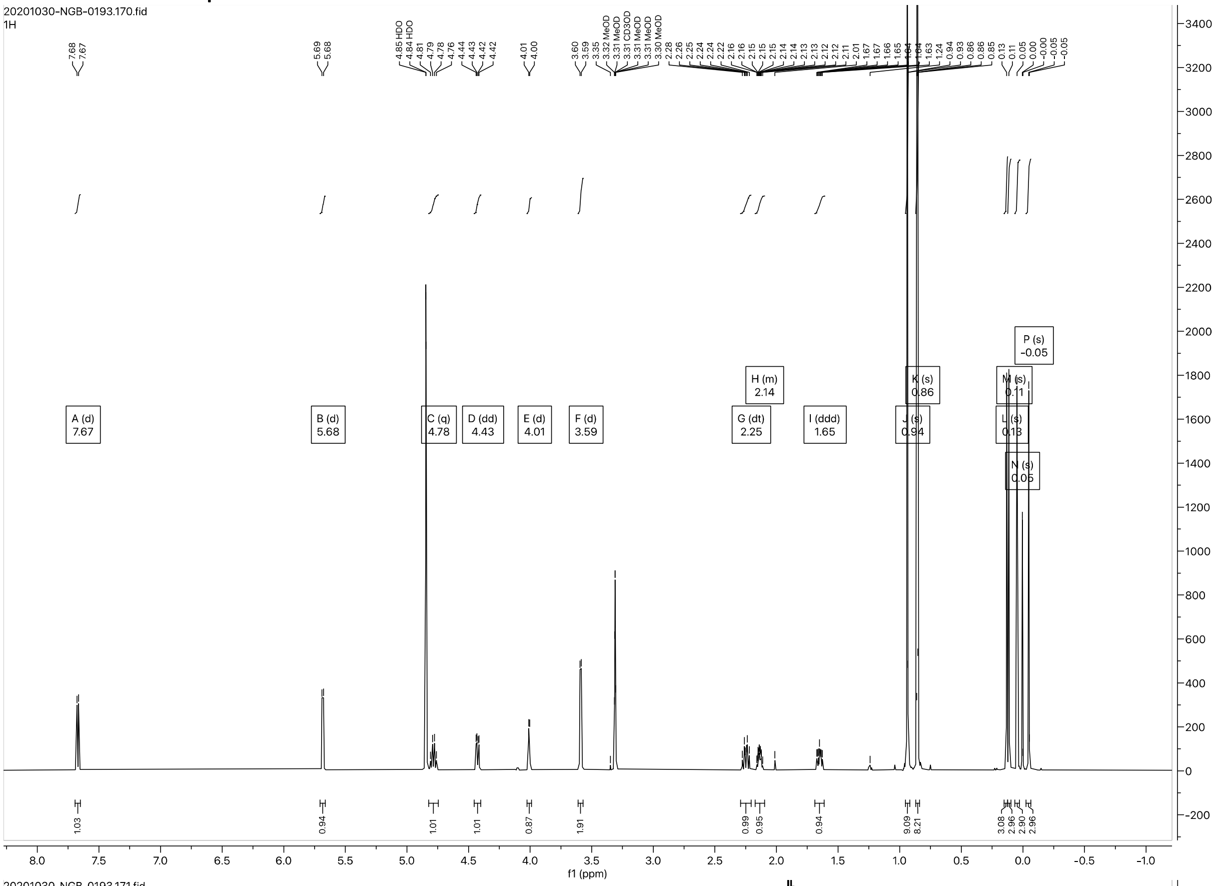

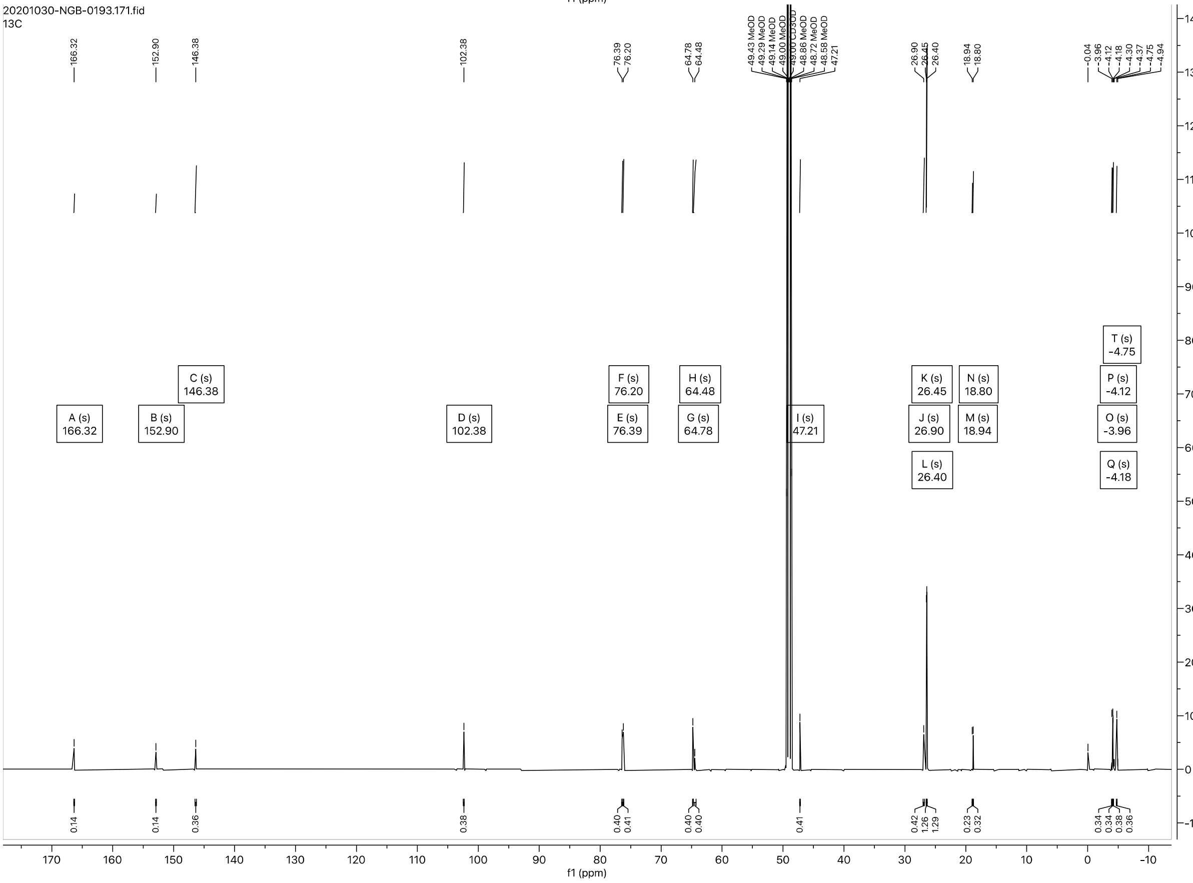

2.3- NMR spectra **17**.

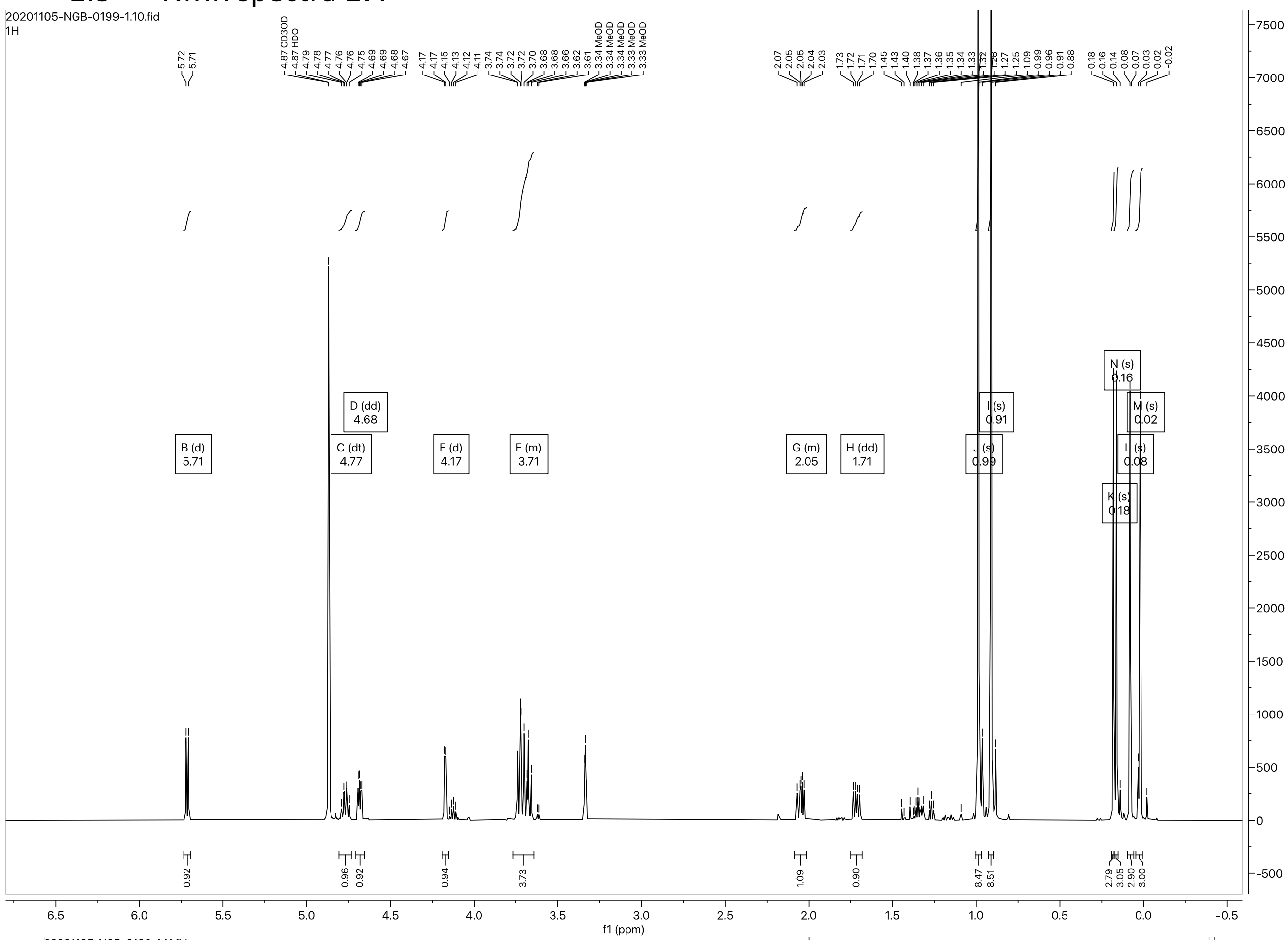

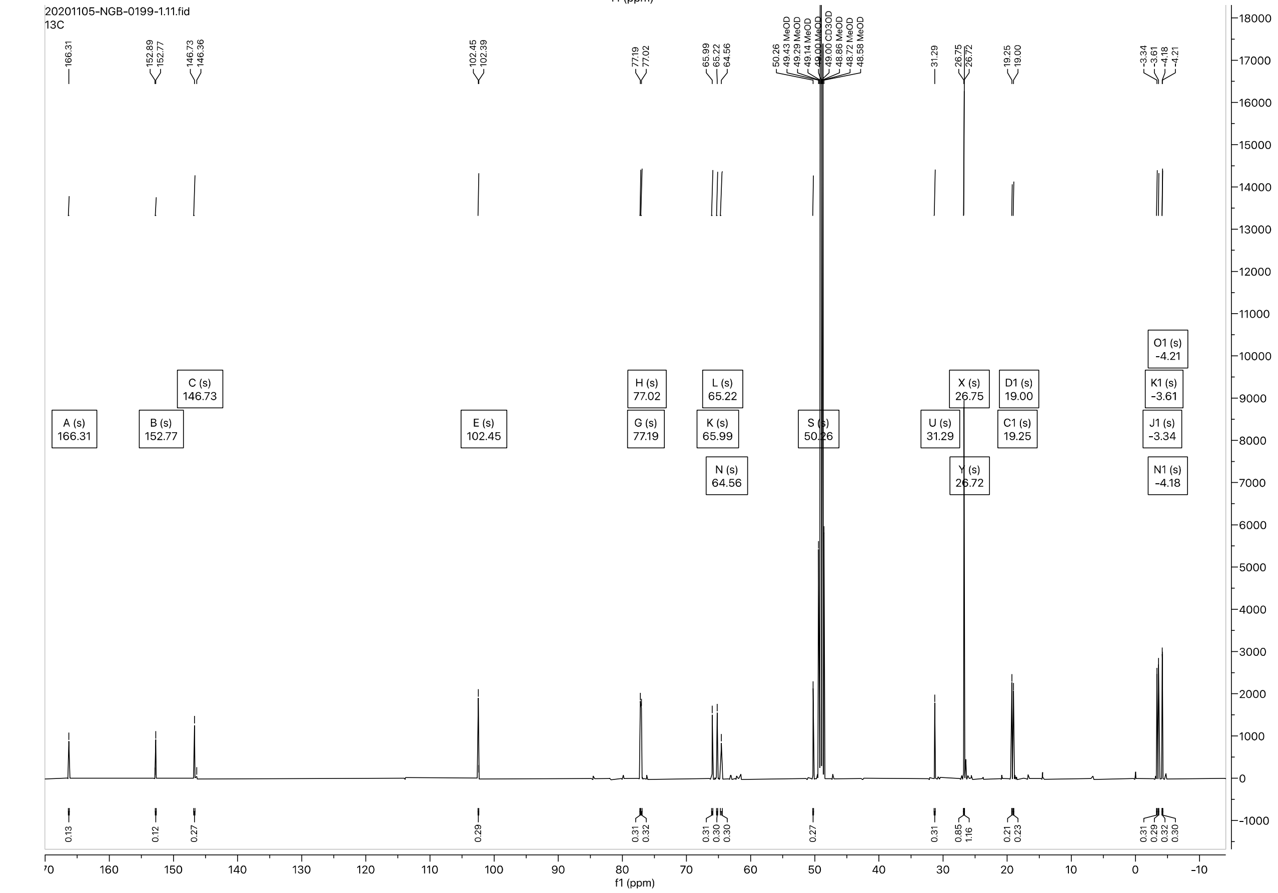

2.4- NMR spectra **20**.

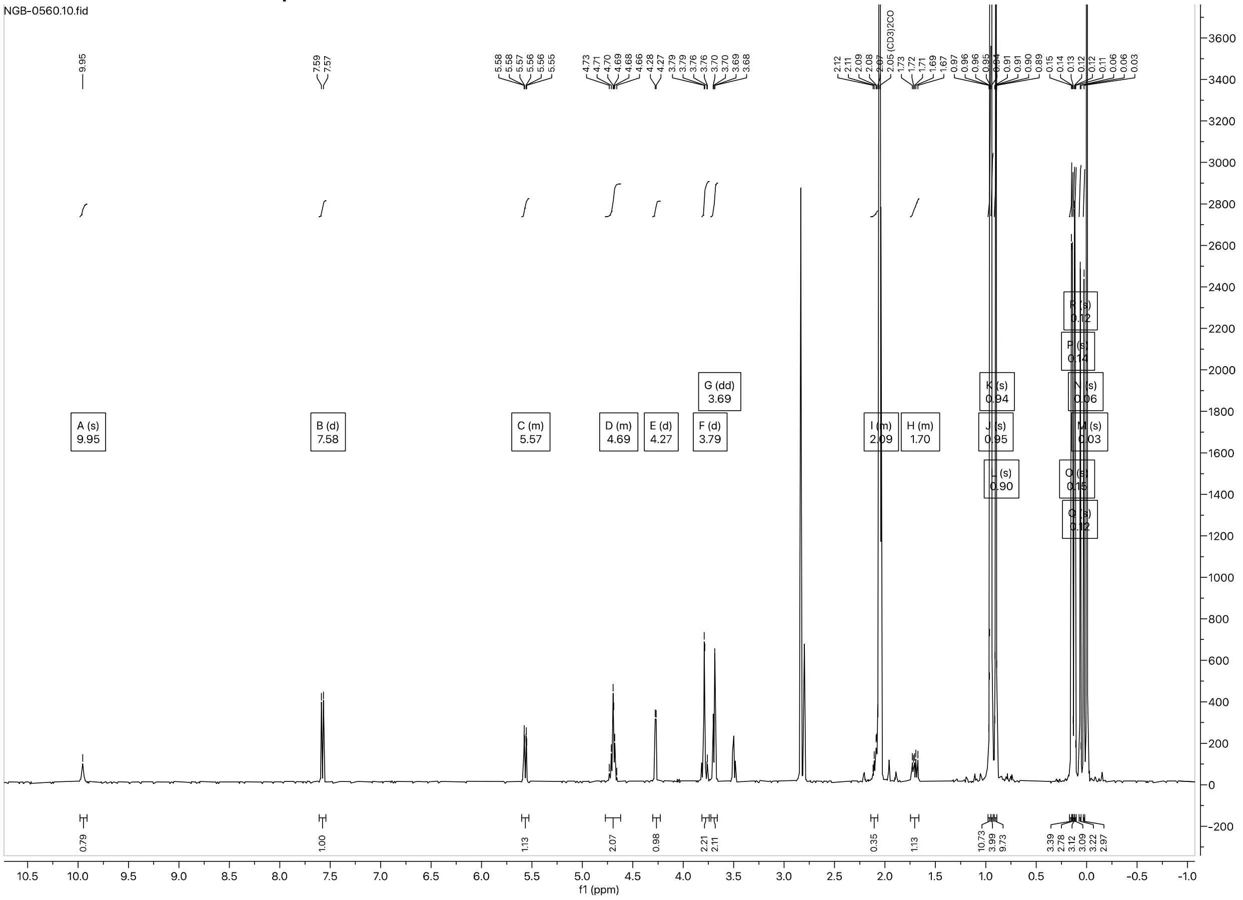

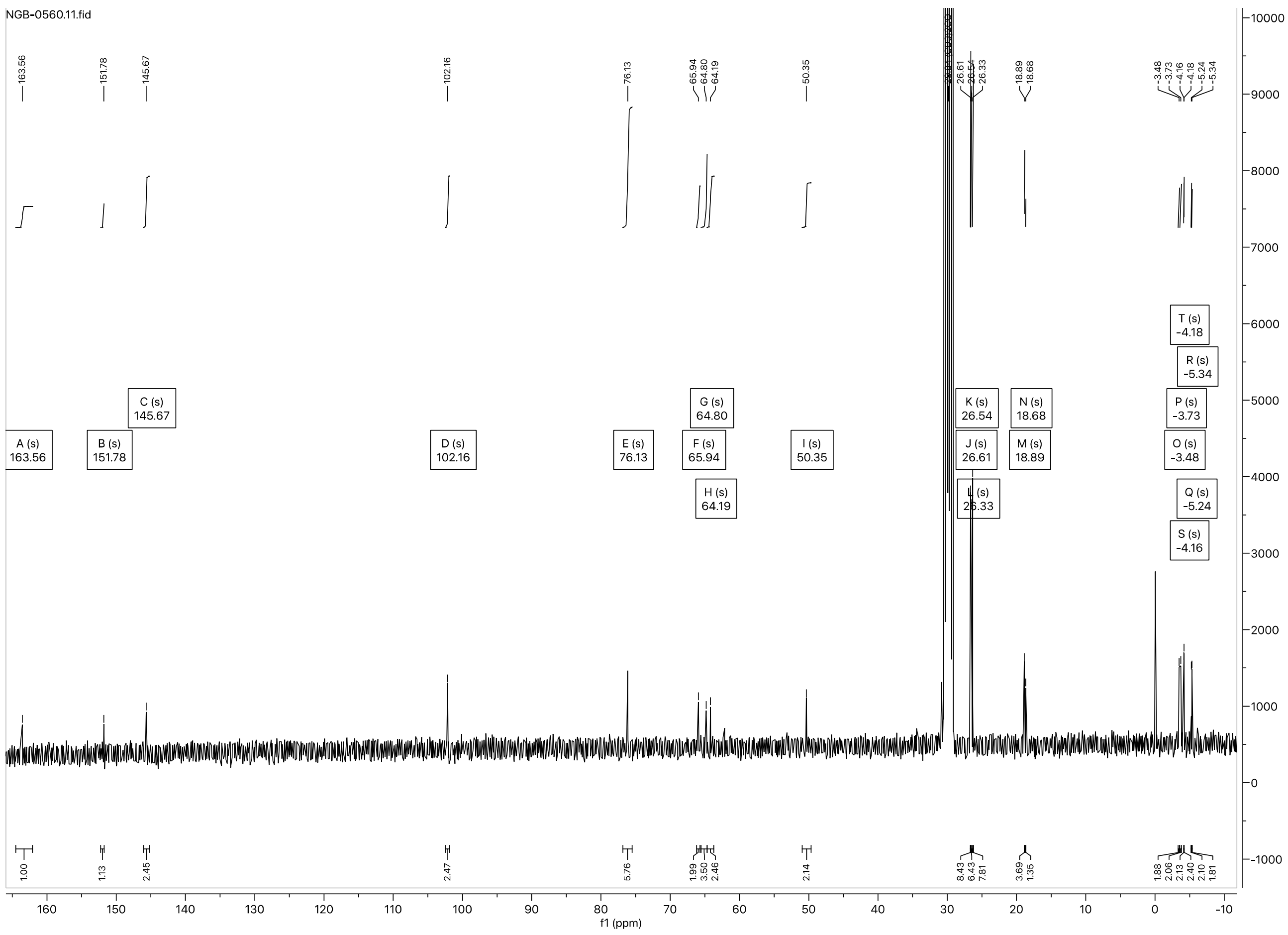

3- Synthesis of 4'-modified nucleoside analogs **9, 10** and **11**).

3.1- Experimental procedures and data.

Procedure for the synthesis of 1-((1*R*,2*S*,3*R*,4*S*)-4-ethynyl-2,3-dihydroxy-4-(hydroxymethyl)cyclopentyl)pyrimidine-2,4(1*H*,3*H*)-dione (9). Under argon, Dess-Martin periodinane (0.16 g, 0.37 mmol) was added to a solution of (**20**) (0.19 g, 0.31 mmol) in dichloromethane (2.5 mL) and pyridine (0.2 mL, 2.48 mmol) at 0 °C. The reaction mixture was stirred at room temperature for 16 h. The reaction mixture was diluted in dichloromethane (20 mL) and sat. $NaHCO_3$ (20 mL) and the precipitate was filtered off. The aqueous layer was extracted with dichloromethane (5 x 10 mL). Organic phases were combined, washed with brine (40 mL), dried over $MgSO_4$, filtered, and concentrated *in vacuo*. The crude aldehyde (**21**) was purified by flash chromatography (ethyl acetate/hexane 0/100 to 70/30) as a white foam (0.16 g). To a solution of this aldehyde (**21**) (0.16 g, 0.26 mmol) in MeOH (7.4 mL) under nitrogen, was added potassium carbonate (0.11 g, 0.78 mmol). The reaction mixture was cooled down to 0 °C before dropwise addition of 1-(dimethoxyphosphinyl)-2-oxo-1-propanediazonium (0.23 mL, 1.56 mmol). The reaction mixture was stirred at room temperature for 16 h before being concentrated *in vacuo*. The crude product was partially purified by flash chromatography (ethyl acetate/hexane 0/100 to 50/50) to give the crude ethynyl derivative (**22**) (0.16 g). To a solution of this compound (**22**) (0.16 g, 0.26 mmol, 1 eq.) in THF (2.6 mL) was added TBAF (1 M in THF, 1.18 mL, 1.18 mmol) at 0 °C. After 12 h at room temperature, volatiles were removed *in vacuo* and the crude product purified by flash chromatography (methanol/dichloromethane 0/100 to 10/90) to give the title compound (**9**) (39.9 mg, 49% over 3 steps) as a white foam. ^{1}H NMR (400 MHz, MeOD-d_4) δ 7.67 (d, 1H, *J* = 8.0 Hz), 5.69 (d, *J* = 8.0 Hz, 1H), 4.80 (ddd, 1H, *J* = 10.6, 9.0, 8.0 Hz), 4.39 (dd, 1H, *J* = 8.0, 6.0 Hz), 3.98 (d, 1H, *J* = 6.0 Hz), 3.68-3.56 (m, 2H), 2.22 (dd, 1H, *J* = 13.1, 9.1 Hz), 2.11-1.97 (m, 1H). ^{13}C NMR (101 MHz, MeOD-d_4) δ 166.3, 152.8, 145.5, 102.5, 85.3, 74.6, 74.4, 73.8, 67.5, 64.9, 58.3, 35.4. HRMS-ESI (*m*/*z*) $[M+H]^+$ calcd. 267.0903. for $C_{12}H_{15}N_2O_5$: found 267.0908.

Procedure for the synthesis of (1*S*,2*R*,3*S*,4*R*)-4-(2,4-dioxo-3,4-dihydropyrimidin-1(2*H*)-yl)-2,3-dihydroxy-1-(hydroxymethyl)cyclopentane-1-carbonitrile 10 Under argon, Dess-Martin periodinane (0.50 g, 1.18 mmol) was added to a solution of (**20**) (0.61 g, 0.99 mmol) in dichloromethane (7.6 mL) and pyridine (0.64 mL, 7.92 mmol) at 0 °C. The reaction mixture was stirred at room temperature for 16 h. The reaction mixture was diluted in dichloromethane (20 mL) and sat.

$NaHCO_3$ (20 mL) and the precipitate was filtered off. The aqueous layer was extracted with dichloromethane (5 x 20 mL). Organic phases were combined, washed with brine (40 mL), dried over $MgSO_4$, filtered, and concentrated *in vacuo*. The crude aldehyde (**21**) was purified by flash chromatography (ethyl acetate/hexane 0/100 to 70/30) as a white foam (0.6 g). To a solution of the 5′-aldehyde (**21**) (0.6 g, 0.98 mmol) in pyridine (5.1 mL) was added NH_2OH. HCl (0.34 mg, 4.9 mmol). After 2 h at room temperature, the volatiles were removed *in vacuo*. The residue was dissolved in ethyl acetate (50 mL), washed with water (15 mL) and brine (15 mL). The organic layer was dried over $MgSO_4$, filtered, and concentrated *in vacuo* to dryness. Crude residue (**23**) (0.63 g) was dissolved in toluene (14.3 mL) before addition of the Burgess reagent (1.19 g, 5 mmol). The reaction was then stirred at 110 °C for 2 h. Volatiles were removed under vacuum ad the residue was dissolved in ethyl acetate (50 mL). The organic layer was washed with sat. $NaHCO_3$ (15 mL), brine (15 mL), dried over $MgSO_4$, filtered, and concentrated *in vacuo*. The crude product was partially purified by flash chromatography (ethyl acetate/hexane 0/100 to 50/50) to give the desired 4′-cyano compound. To a solution of the crude 4′-cyano compound (0.4 g) in THF (6.5 mL) was added TBAF (1M in THF, 2.6 mL, 2.6 mmol) at 0 °C. After 12 h at room temperature, the volatiles were removed under in vacuo and the crude product was purified by flash chromatography (methanol/dichloromethane 0/100 to 10/90). Then TBAF solid salt was washed with methanol (2x1 mL) to remove TBAF traces to give the title compound (**10**) (0.1 g, 44% over 4 steps) as a white foam. ^{1}H NMR (400 MHz, D_2O-*d₂*) δ 7.62 (d, 1H, *J* = 8.0 Hz), 5.82 (d, 1H, *J* = 8.0 Hz), 4.67 (q, 1H, *J* = 9.3 Hz), 4.50 (dd, 1H, *J* = 8.3, 5.4 Hz), 4.17 (d, 1H, *J* = 5.4 Hz), 3.86-3.74 (m, 2H), 2.58 (dd, 1H, *J* = 14.2, 9.3 Hz), 2.11 (dd, 1H, *J* = 14.2, 10.3 Hz). ^{13}C NMR (101 MHz, D_2O-*d₂*) δ 166.3, 152.0, 145.4, 121.2, 101.9, 71.9, 71.6, 64.0, 63.1, 48.1, 31.3. HRMS-ESI (m/z) [M+H]+ calcd. 268.0855. for $C_{11}H_{14}N_3O_5$: found 268.0933.

Procedure for the synthesis of 1-((1*R*,2*S*,3*R*,4*S*)-2,3-bis((*tert*-butyldimethylsilyl)oxy)-4-(((*tert*-butyldimethylsilyl)oxy)methyl)-4-(chloromethyl)cyclopentyl)pyrimidine-2,4(1*H*,3*H*)-dione (24). A mixture of (**20**) (0.056 g, 0.071 mmol), Ph_3P (0.048 g, 0.18 mmol), and CCl_4 (0.02 mL, 0.18 mmol) in DCE (1 mL) was heated at 130 °C under microwave irradiation under nitrogen for 40 min. The volatiles were removed *in vacuo* and the crude product was purified by flash chromatography (ethyl acetate/hexane 0/100 to 50/50) to give the title compound (**24**) (38.6 mg, 67%) as a white foam. ^{1}H NMR (400 MHz, Me_2CO-*d₆*) δ 10.05 (s, 1H), 7.56 (d, 1H, *J* = 8.0 Hz), 5.57 (dd, 1H, *J* = 8.0, 2.1 Hz), 4.87 (dd, 1H, *J* = 9.2, 3.8 Hz), 4.59 (dt, 1H, *J* = 10.6, 8.8 Hz), 4.23 (d, 1H, *J* = 3.7 Hz, 1H), 3.87-3.77 (m, 4H), 2.14-2.06 (m, 1H), 1.84 (dd, 1H, *J* = 14.0, 8.5 Hz), 0.97 (s, 9H), 0.95 (s, 9H), 0.90 (s, 9H), 0.18 (s, 3H), 0.14 (s, 6H), 0.13 (s, 3H), 0.06 (s, 3H), 0.01 (s, 3H). ^{13}C NMR (101 MHz, Me_2CO-*d₆*) δ 163.6, 151.8, 146.7, 102.1, 76.3, 75.0, 65.6, 64.5, 49.9, 49.6, 30.7, 26.7, 26.5, 26.2, 18.9, 18.8, 18.7, -3.3, -3.6, -4.0, -4.3, -5.2, -5.3. HRMS-ESI (*m/z*) [M+H]$^+$ calcd. 633.3264. for $C_{29}H_{58}ClN_2O_5Si_3$: found 633.3349.

Procedure for the synthesis of 1-((1*R*,2*S*,3*R*,4*S*)-4-(((*tert*-butyldimethylsilyl)oxy)methyl)-4-(chloromethyl)-2,3-dihydroxycyclopentyl)pyrimidine-2,4(1*H*,3*H*)-dione (11). TBAF (1M in THF, 0.4 mL, 0.4 mmol) was added at 0 °C to a solution of (**24**) (0.065 mg, 0.1 mmol) in THF (1.1 mL). After 12 h at room temperature, the volatiles were removed *in vacuo* and the crude product was purified by flash chromatography (methanol /dichloromethane 0/100 to 10/90). to give the title compound (**11**) (26.4 mg, 89%) as a white foam. ^{1}H NMR (400 MHz, MeOD-*d₄*) δ 7.68 (d, 1H, *J* = 8.0 Hz), 5.68 (d, 1H, *J* = 7.9 Hz), 4.72 (q, 1H, *J* = 9.8 Hz), 4.50 (dd, 1H, *J* = 10.1, 4.6 Hz), 3.95 (d, 1H, *J* = 4.6 Hz), 3.86 (d, 1H, *J* = 10.9 Hz), 3.78-3.57 (m, 4H), 2.04 (dd, 1H, *J* = 14.0, 9.9 Hz), 1.70-1.62 (m, 1H).^{13}C NMR (101 MHz, MeOD-*d₄*) δ 166.3, 153.0, 145.3, 102.4, 74.8, 74.6, 65.3, 63.2, 49.4, 48.0, 32.8. HRMS-ESI (m/z) [M+H]+ calcd. 291.0669. for $C_{11}H_{16}ClN_2O_5$: found 291.0748.

3.2- NMR spectra **9**.

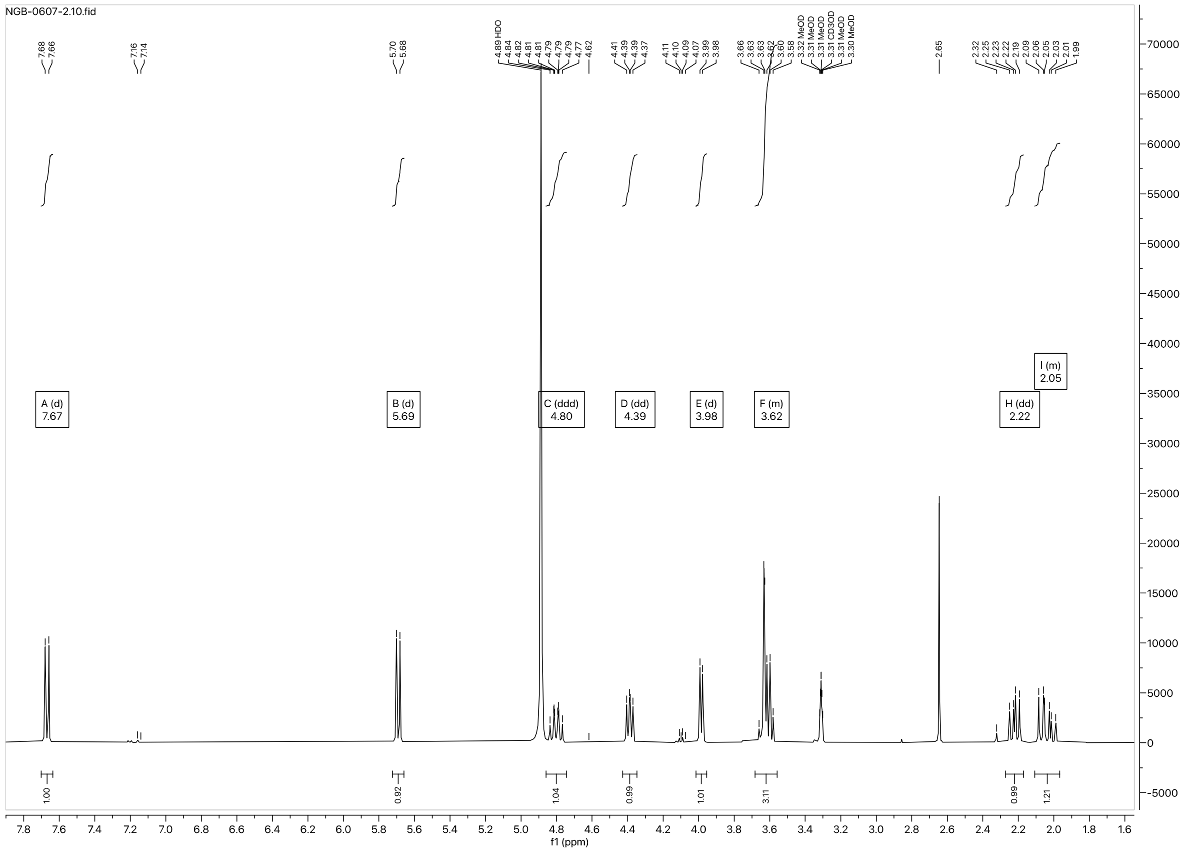

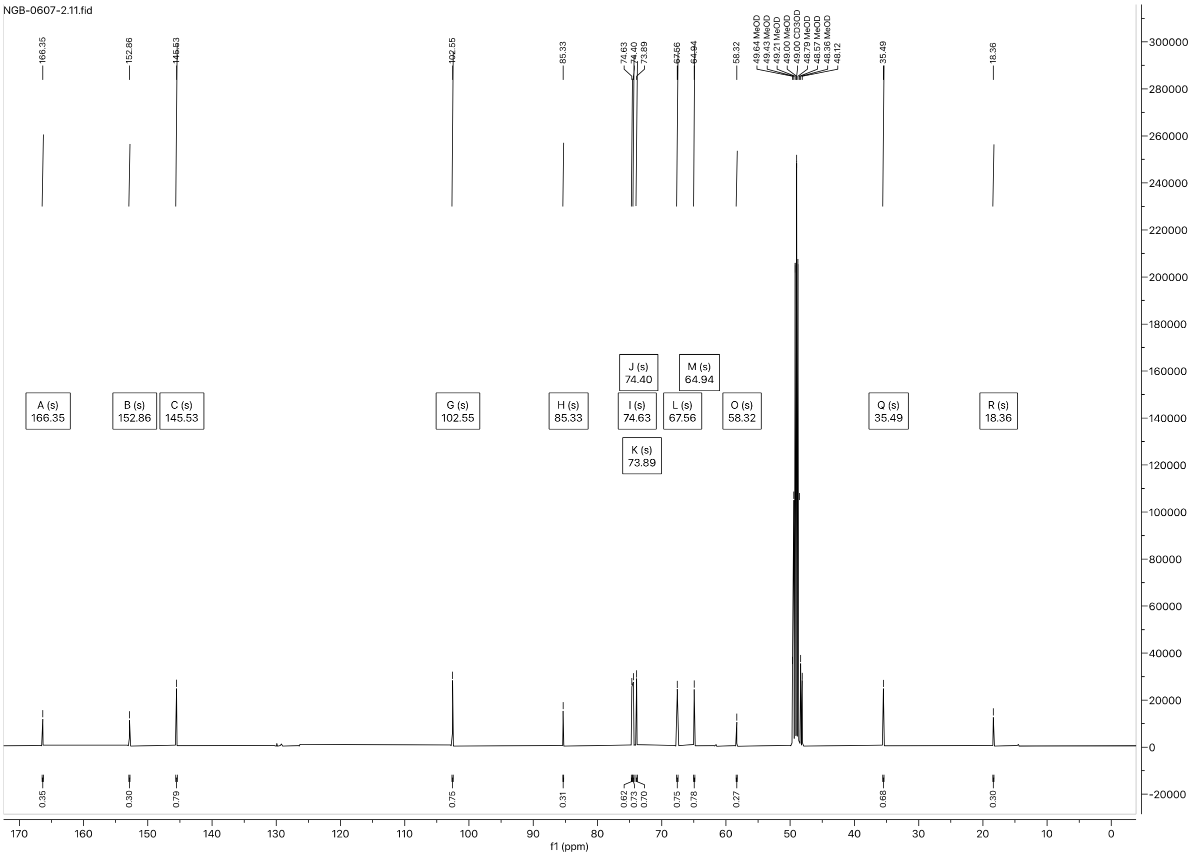

3.3- NMR spectra **10**.

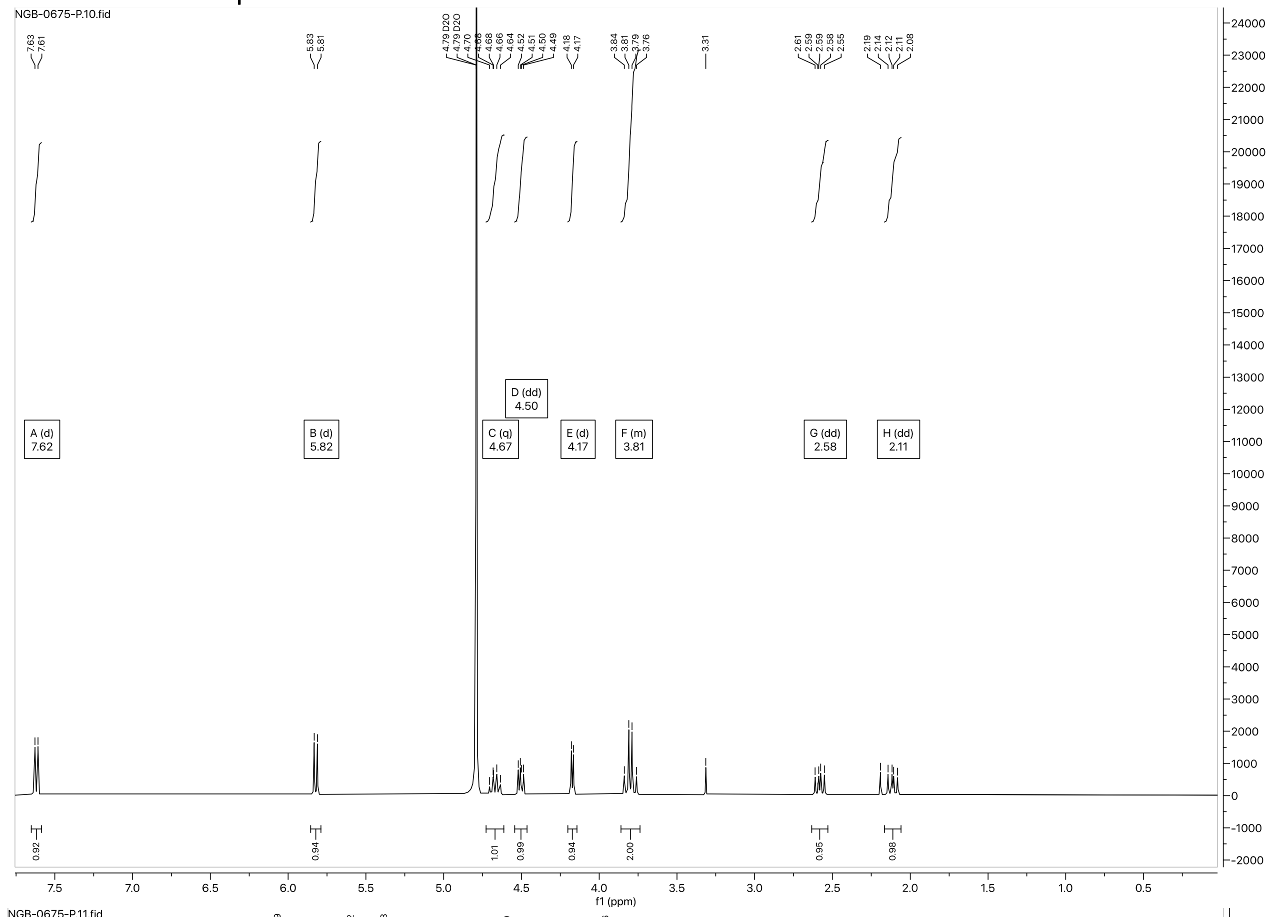

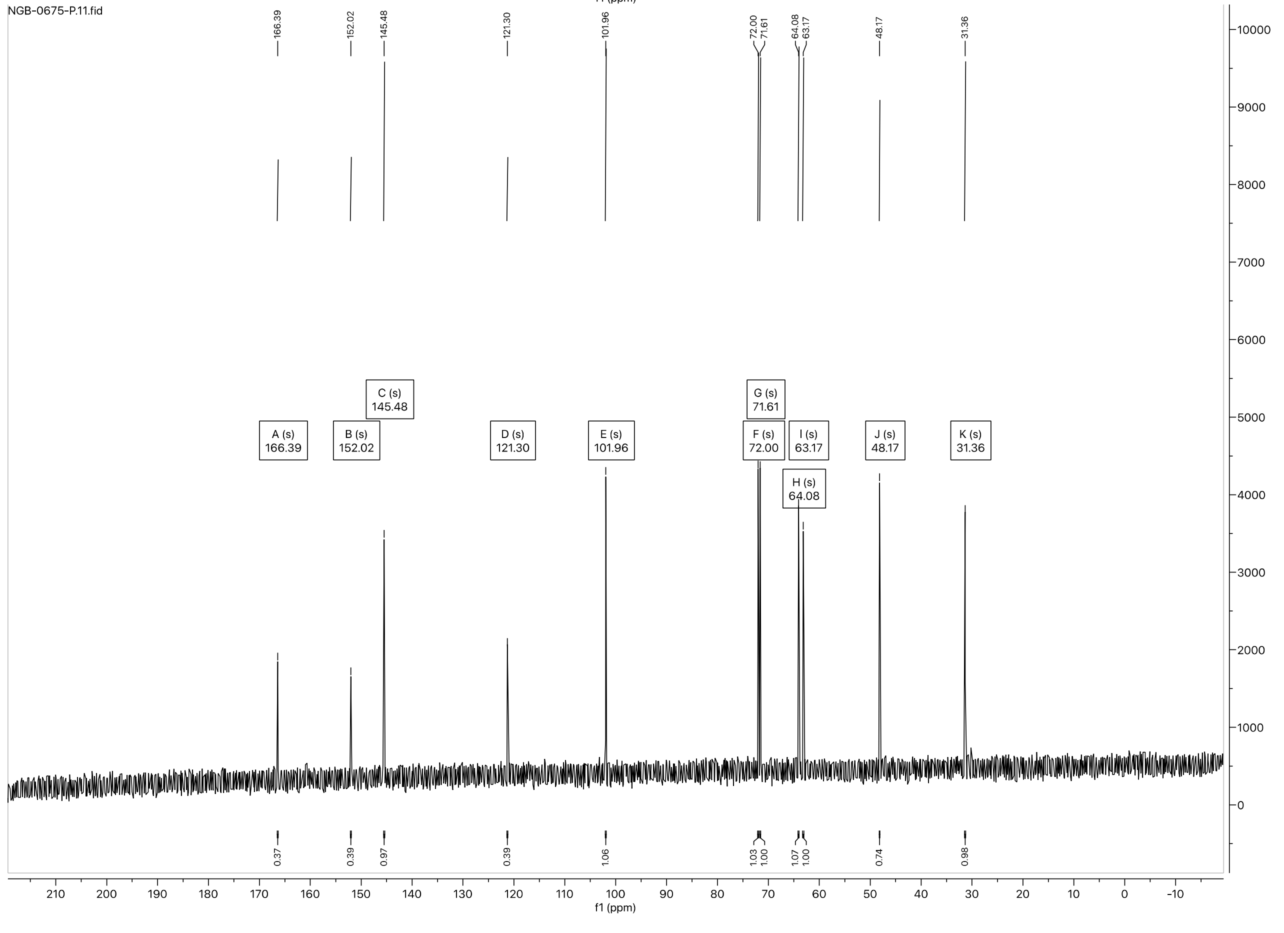

3.4- NMR spectra **24**.

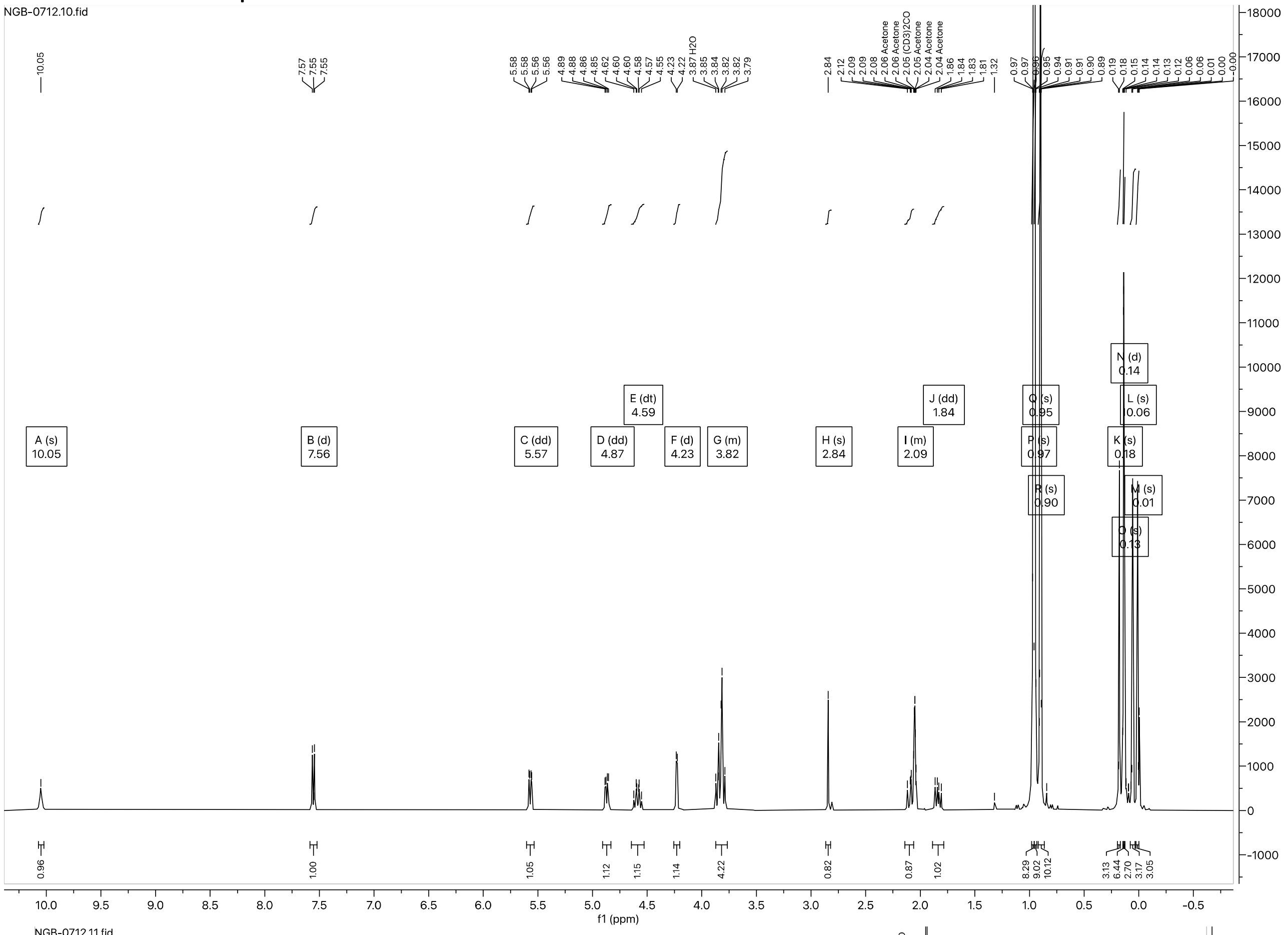

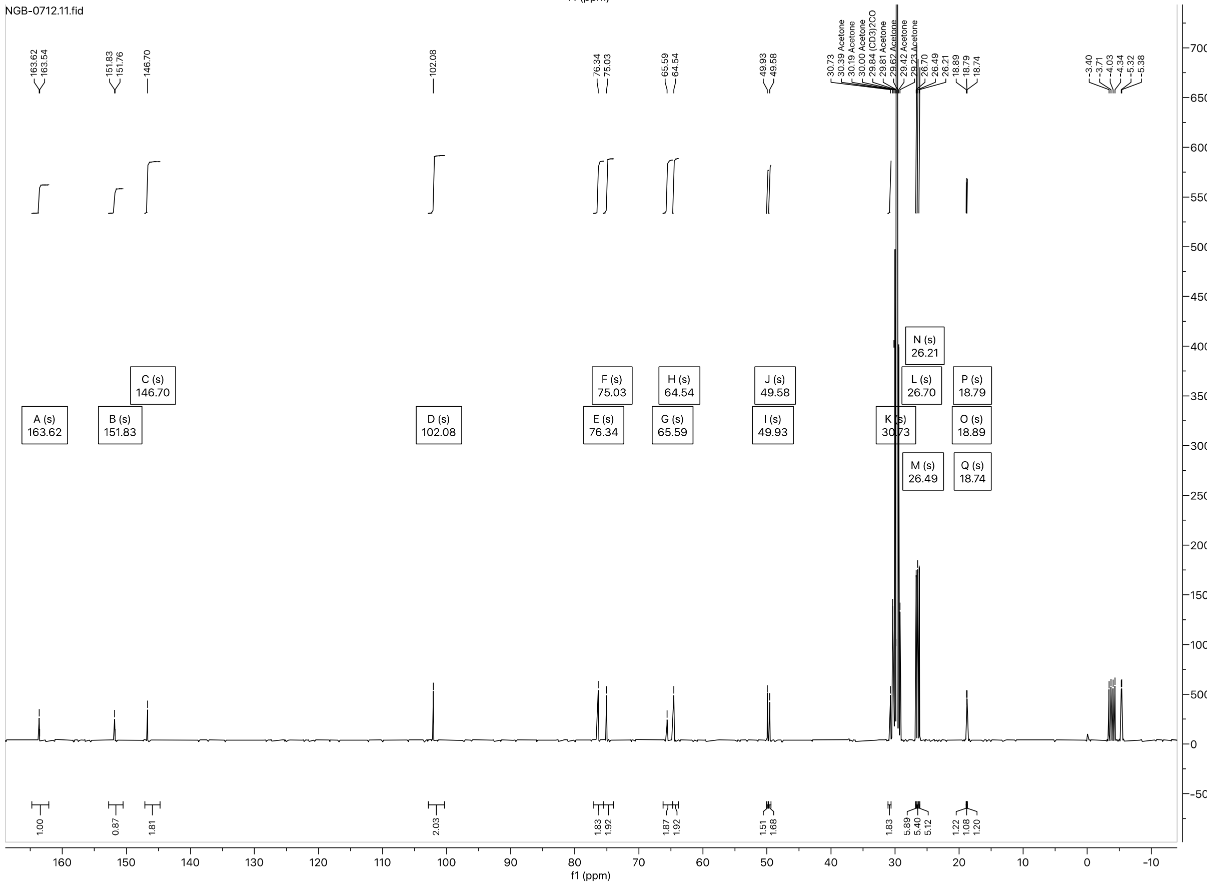

3.5- NMR spectra **11**.

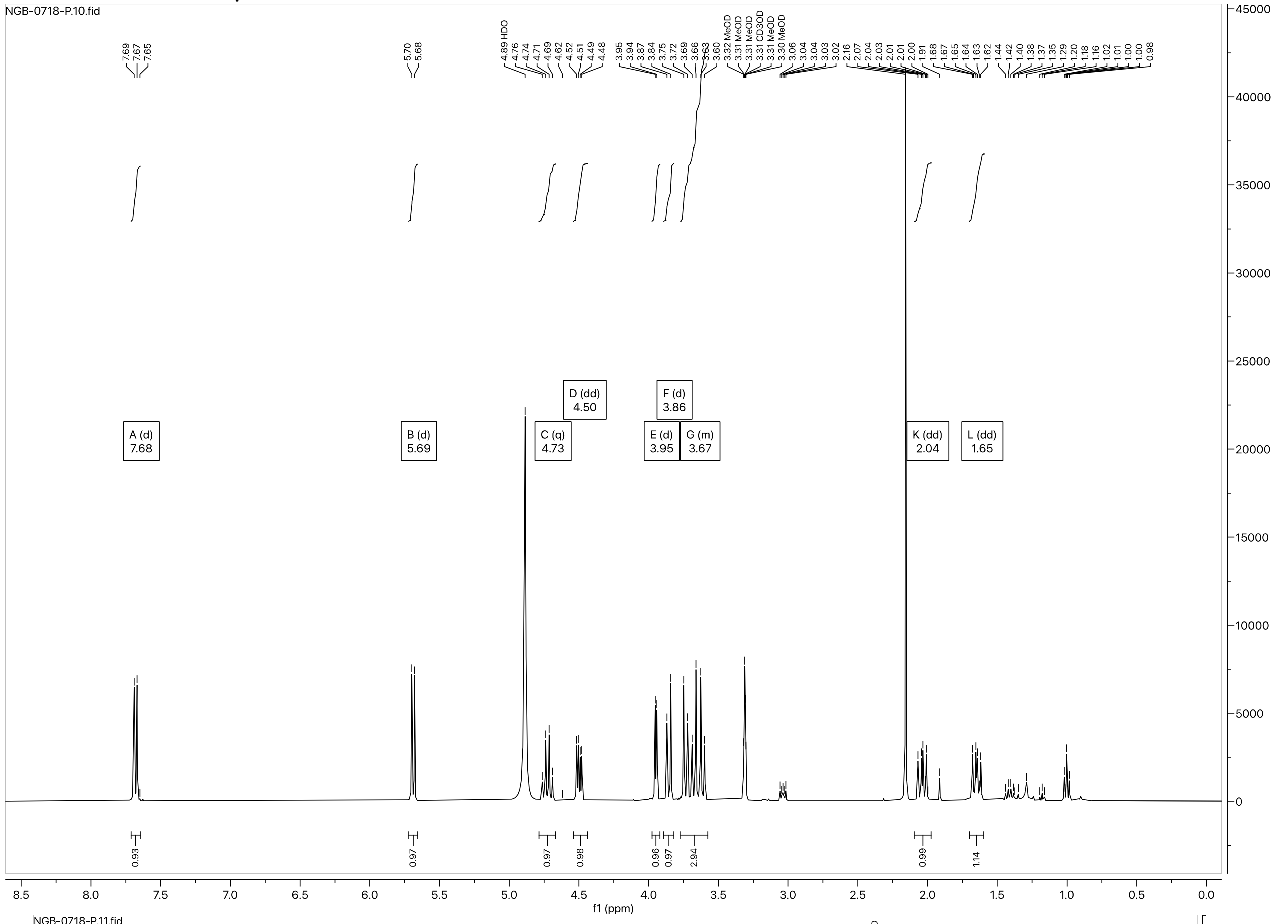

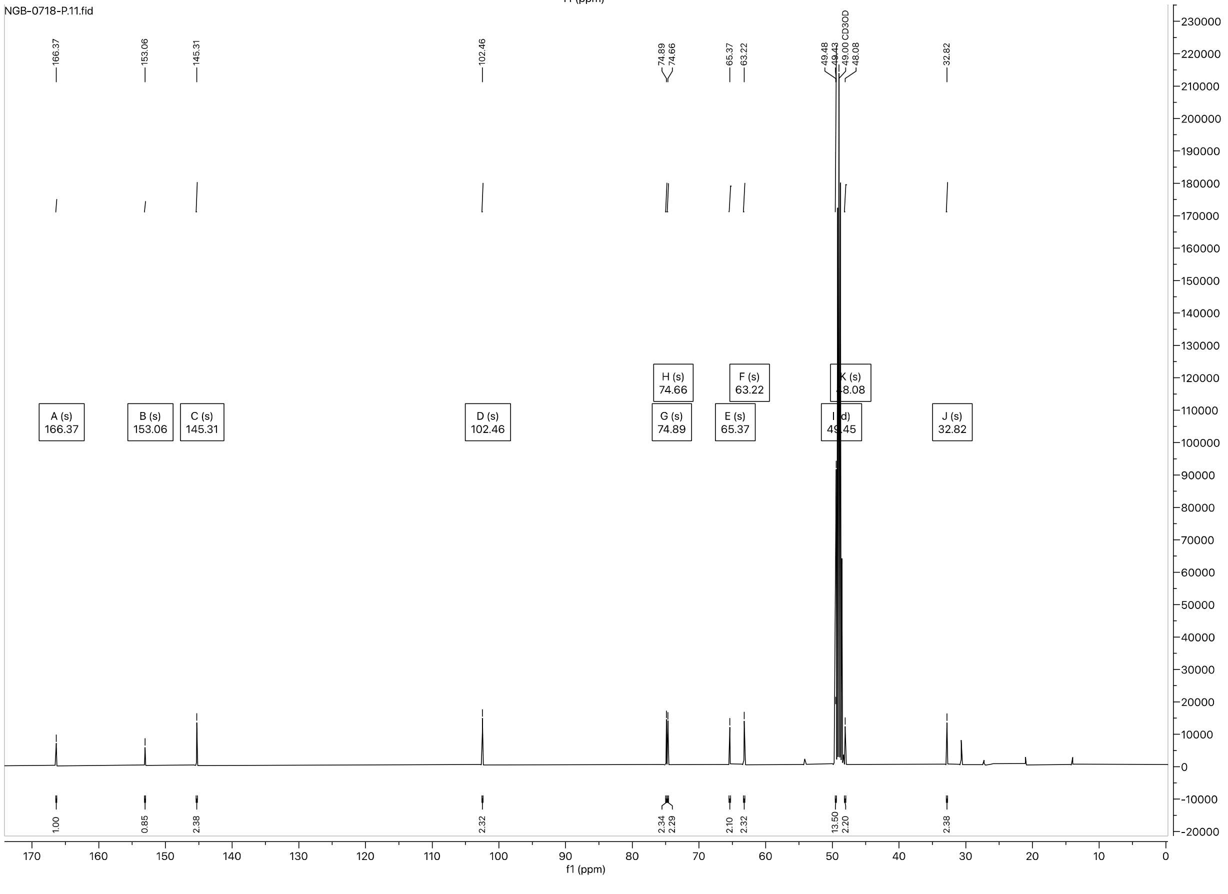

4- Synthesis of monophosphate prodrugs **12**, **13** and **14**.

4.1- Experimental procedures and data.

Procedure for the synthesis of 1-((3a*S*,4*R*,6*S*,6a*R*)-6-ethynyl-6-(hydroxymethyl)-2,2-dimethyltetrahydro-4*H*-cyclopenta[*d*][1,3]dioxol-4-yl)pyrimidine-2,4(1*H*,3*H*)-dione (25). To a solution of (**9**) (60 mg, 0.23 mmol) in acetone (4.5 mL) was added dimethoxypropane (0.03 mL, 0.23 mmol) and conc. sulfuric acid (0.002 mL, 0.046 mmol) at room temperature. The resulting reaction mixture was stirred for 3 h before addition of solid $NaHCO_3$ (pH = 7). The resulting mixture was filtered off and concentrated *in vacuo* to dryness. The residue purified by flash chromatography (methanol/dichloromethane 0/100 to 10/90) to give the title compound (**25**) (45 mg, 65 %) as a white foam. ^{1}H NMR (400 MHz, MeOD-*d4*) δ 7.70 (d, 1H, *J* = 8.0 Hz), 5.70 (d, *J* = 7.9 Hz, 1H), 5.01 (ddd, 1H, *J* = 12.2, 7.3, 4.9 Hz), 4.92-4.85 (m, 1H), 4.63 (d, 1H, *J* = 7.5 Hz), 3.68-3.56 (m, 2H), 2.75 (s, 1H), 2.47 (t, 1H, *J* = 12.4 Hz), 2.21 (dd, 1H, *J* = 12.4, 7.3 Hz), 1.59 (s, 3H), 1.31 (s, 3H). ^{13}C NMR (101 MHz, MeOD-*d4*) δ 166.2, 152.5, 145.3, 115.9, 102.8, 85.1, 84.0, 82.2, 75.8, 66.9, 63.8, 40.0, 26.6, 25.6. HRMS-ESI (*m/z*) $[M+H]^+$ calcd. 307.1216. for $C_{15}H_{19}N_2O_5$: found 307.1293.

Procedure for the synthesis of isopropyl ((((1*S*,2*R*,3*S*,4*R*)-4-(2,4-dioxo-3,4-dihydropyrimidin-1(2*H*)-yl)-1-ethynyl-2,3-dihydroxycyclopentyl)methoxy)(phenoxy)phosphoryl)-*L*-alaninate (12). Under inert atmosphere, the isopropylidene derivative (**17**) (45 mg, 0.15 mmol) was dissolved in anhydrous THF (0.6 mL) containing 4 Å molecular thieves. The reaction mixture was cooled down to 0 °C before dropwise addition of *t*-BuMgCl (1M in THF, 0.47 mL, 0.47 mmol). The reaction was stirred for 30 min at 0 °C and then for 30 min at room temperature. The reaction was then cooled down to 0 °C before dropwise addition of a solution of isopropyl ((*S*)-(perfluorophenoxy)-(phenoxy)phosphoryl)-*L*-alaninate (0.10 g, 0.23 mmol) in dry THF (0.46 mL). The reaction was then stirred overnight at room temperature before dropwise addition of HCl (12 M, 0.5 mL) at 0 °C. The mixture was stirred at room temperature for 4 h and then quenched with ammonia in methanol (10 mL), then volatiles were removed *in vacuo.* The residue was purified by flash chromatography (methanol/dichloromethane 0/100 to 15/85) to give the title compound (**12**) (26 mg, 34% over 2 steps) as a white foam. ^{1}H NMR (400 MHz, MeOD-*d4*) δ 7.53 (d, 1H, *J* = 8.0 Hz), 7.37 (t, 2H, *J* = 8.0 Hz), 7.27 (d, 2H, *J* = 7.6 Hz), 7.20 (t, 1H, *J* = 7.6 Hz), 5.65 (d,1 H, *J* = 8.0 Hz), 4.99 (hept, 1H, *J* = 6.4 Hz), 4.75-4.65 (m, 1H), 4.35 (t, 1H, *J* = 6.8 Hz), 4.15 (dd, 2H, *J* = 6.6, 5.1 Hz), 4.02 (d, 1H, *J* = 6.4 Hz), 3.96-4.01 (m, 1H), 2.74 (s, 1H), 2.21 (dd, 1H, *J* = 13.0, 8.8 Hz), 2.14-2.06 (m, 1H), 1.36 (d, *J* = 7.2 Hz, 3H), 1.24 (dd, *J* = 6.3, 1.3 Hz, 6H). ^{13}C NMR (101 MHz, MeOD-*d4*) δ 174.4 (d, *J* = 5.4 Hz), 166.3, 152.6, 152.1 (d, *J* = 7.1 Hz), 146.0, 130.8, 126.2, 121.6 (d, *J* = 4.6 Hz), 102.4, 83.6, 75.7, 73.7, 73.2, 70.5 (d, *J* = 5.5 Hz), 70.2, 66.0, 51.6, 47.1 (d, *J* = 8.9 Hz), 35.1, 21.9 (d, *J* = 9.2 Hz), 20.5 (d, *J* = 6.4 Hz). ^{31}P NMR (162 MHz, MeOD-*d4*) δ 3.17. HRMS-ESI (*m/z*) $[M+H]^+$ calcd. 536.17209. for $C_{24}H_{31}N_3O_9P$: found 536.1807.

Procedure for the synthesis of isopropyl ((((1*S*,2*R*,3*S*,4*R*)-1-cyano-4-(2,4-dioxo-3,4-dihydropyrimidin-1(2*H*)-yl)-2,3-dihydroxycyclopentyl)methoxy)(phenoxy)phosphoryl)-*L*-alaninate (13). Under inert atmosphere, (**10**) (96.1 mg g, 0.36 mmol) was dissolved into a mixture of anhydrous THF (1.2 mL) and anhydrous DMF (3 ml) containing 4 Å molecular thieves. The mixture was cooled down to 0 °C before dropwise addition of *t*-BuMgCl (1M in THF, 0.72 mL, 0.72 mmol). The reaction was then stirred for 30 min at 0 °C and then for 30 min at room temperature. The reaction mixture was cooled down to 0 °C before dropwise addition of a solution of isopropyl ((*S*)-(perfluorophenoxy)-(phenoxy)phosphoryl)-*L*-alaninate (0.16 g, 0.34 mmol) in THF (1.2 mL). The reaction was stirred overnight at 0 °C and then diluted with sat. $NaHCO_3$ (25 mL) and extracted with ethyl acetate (3 x 20 mL). The organic layers were combined, washed with brine (20 mL), dried over $MgSO_4$, filtered, and concentrated *in vacuo* to dryness. The residue was purified by flash chromatography (methanol/dichloromethane 0/100 to 10/90) to give the title compound (**13**) (24.7 mg, 13%) as a white foam. ^{1}H NMR (400 MHz, MeOD-*d4*) δ 7.56 (d, *J* = 7.9 Hz, 1H), 7.42-7.34 (m, 2H), 7.28 (dt, *J* = 8.7, 1.3 Hz, 2H), 7.26-7.16 (m, 1H), 5.65 (d, *J* = 7.9 Hz, 1H), 4.99 (hept, 1H, *J* = 6.2 Hz), 4.60-4.47 (m, 2H), 4.31 (ddd, 2H, *J* = 49.5, 10.5, 5.1 Hz), 4.12 (d, *J* = 4.6 Hz, 1H), 3.95 (dq, *J* = 10.0, 7.1 Hz, 1H), 2.61-2.47 (m, 1H), 2.27-2.13 (m, 1H), 1.42-1.33 (m, 3H), 1.24 (td, *J* = 6.8, 6.3, 3.4 Hz, 9H). ^{13}C NMR (101 MHz, MeOD-*d4*) δ 174.3 (d, *J* = 5.4 Hz), 166.4, 152.5, 152.0 (d, *J* = 7.0 Hz), 146.8, 130.8, 126.3 (d, *J* = 1.4 Hz), 121.5 (d, *J* = 4.7 Hz), 120.9, 102.3, 73.2, 73.0, 70.2, 69.1 (d, *J* = 4.9 Hz), 66.0, 51.6, 50.7, 33.0, 21.9 (d, *J* = 6.9 Hz), 20.4 (d, *J* = 6.5 Hz). ^{31}P NMR (162 MHz, MeOD-*d4*) δ 3.00. HRMS-ESI (*m/z*) $[M+H]^+$ calcd. 537.1672 for $C_{23}H_{29}N_4O_9P$: found 536.1807.

Procedure for the synthesis of isopropyl ((((1*S*,2*R*,3*S*,4*R*)-1-(chloromethyl)-4-(2,4-dioxo-3,4-dihydropyrimidin-1(2*H*)-yl)-2,3-dihydroxycyclopentyl)methoxy)(phenoxy)phosphoryl)-*L*-alaninate (14). Under inert atmosphere, (**11**) (0.12 g, 0.41 mmol) was dissolved into anhydrous THF (1.5 mL) containing 4 Å molecular thieves. The mixture was cooled down to 0 °C before dropwise addition of *t*-BuMgCl (1M in THF, 0.82 mL, 0.82 mmol). The reaction was then stirred for 30 min at 0 °C and then for 30 min at room temperature. The reaction mixture was cooled down to 0 °C before dropwise addition of a solution of isopropyl ((*S*)-(perfluorophenoxy)-(phenoxy)phosphoryl)-*L*-alaninate (0.18 g, 0.38 mmol) in THF (1.4 mL). The reaction was stirred overnight at 0 °C and then diluted with sat. $NaHCO_3$ (25 mL) and extracted with ethyl acetate (3 x 20

mL). The organic layers were combined, washed with brine (20 mL), dried over $MgSO_4$, filtered, and concentrated *in vacuo* to dryness. The residue was purified by flash chromatography (methanol/dichloromethane 0/100 to 10/90) to give the title compound (**14**) (10.3 mg, 5%) as a white foam. ^{1}H NMR (400 MHz, MeOD-d_4) δ 7.58 (d, 1H, J = 8.0 Hz), 7.38 (dd, 2H, J = 8.6, 7.2 Hz), 7.26 (dq, 2H, J = 7.8, 1.2 Hz), 7.20 (d, 1H, J = 7.4, 1.1 Hz), 5.65 (d, 1H, J = 7.9 Hz), 4.98 (hept, 1H, J = 6.3 Hz), 4.67-4.56 (m, 1H) 4.50 (d, J = 10.0, 4.6 Hz, 1H), 4.27-4.13 (m, 2H), 3.99-3.87 (m, 2H), 3.86-3.69 (m, 2H), 2.07 (dd, 1H, J = 14.2, 9.7 Hz), 1.73 (dd, 1H, J = 14.2, 9.6 Hz), 1.36 (dd, 3H, J = 7.1, 1.0 Hz), 1.24 (dd, 6H, J = 6.2, 3.1 Hz). ^{13}C NMR (101 MHz, MeOD-d_4) δ 174.4 (d, J = 5.4 Hz), 166.3, 152.8, 152.2 (d, J = 7.0 Hz), 145.8, 130.8, 126.2, 121.4 (d, J = 4.7 Hz), 102.3, 74.2, 73.9, 70.2, 69.2 (d, J = 5.7 Hz), 64.0, 51.7, 47.4, 32.2, 21.95 (d, J = 7.7 Hz), 20.5 (d, J = 6.4 Hz). ^{31}P NMR (162 MHz, MeOD-d_4) δ 3.26. HRMS-ESI (m/z) $[M+H]^+$ calcd 560.1486. for $C_{23}H_{31}ClN_3O_9P$: found 560.1572.

4.2- NMR spectra **25**.

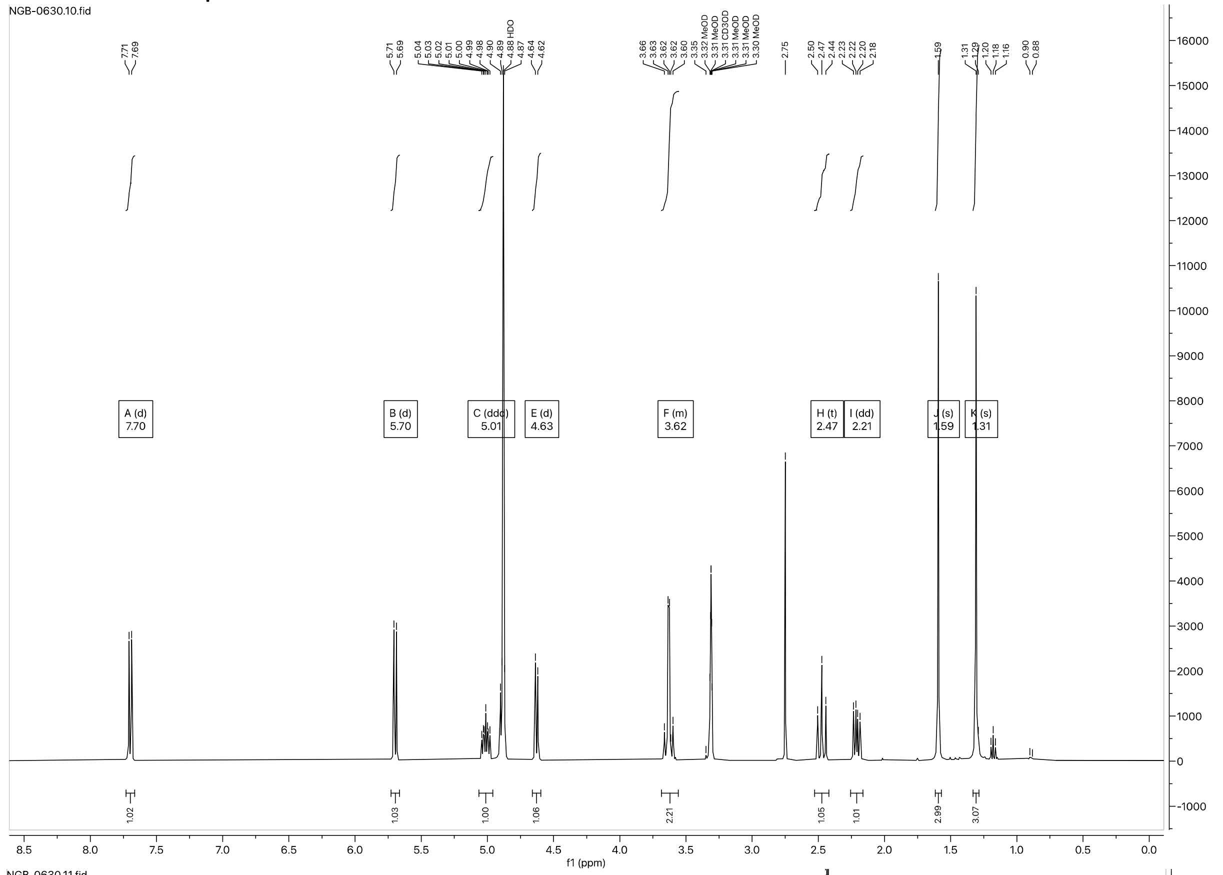

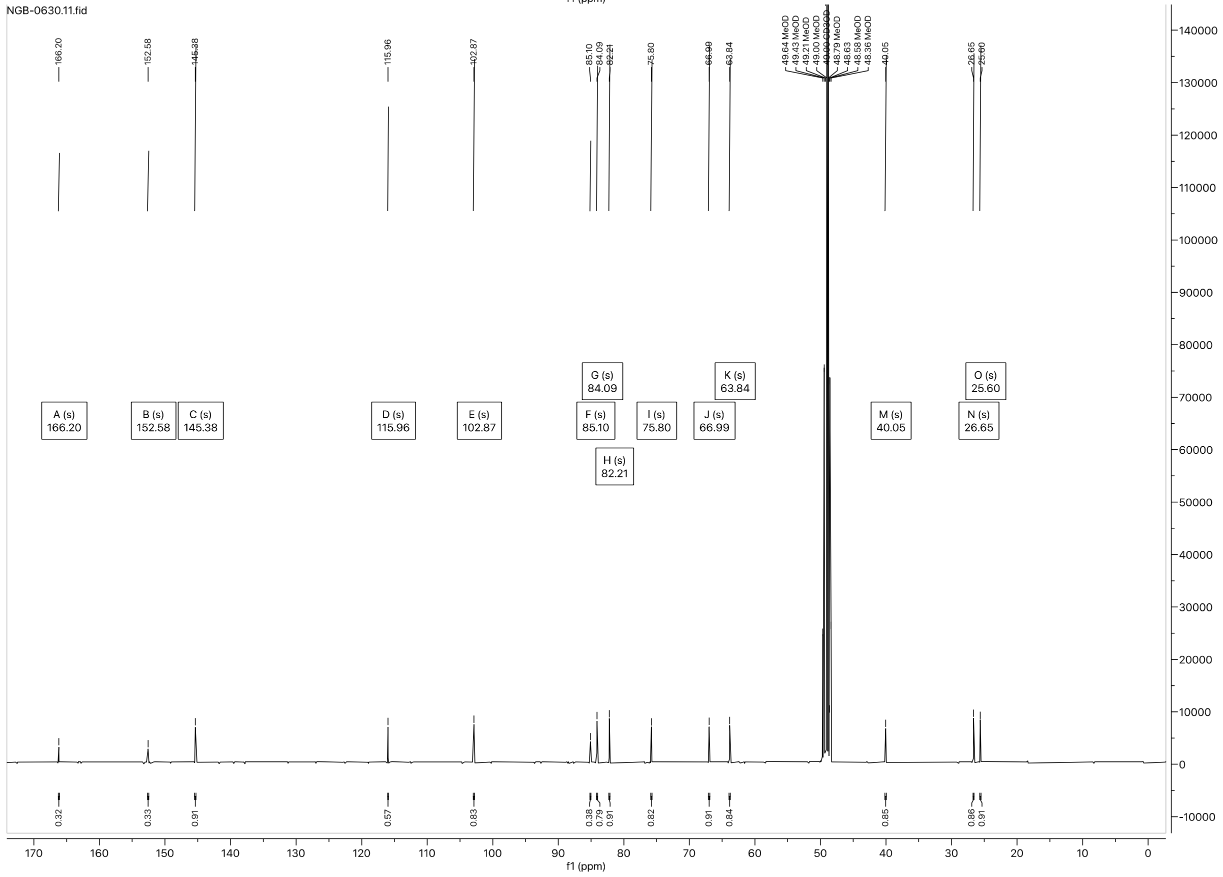

4.3- NMR spectra **12**.

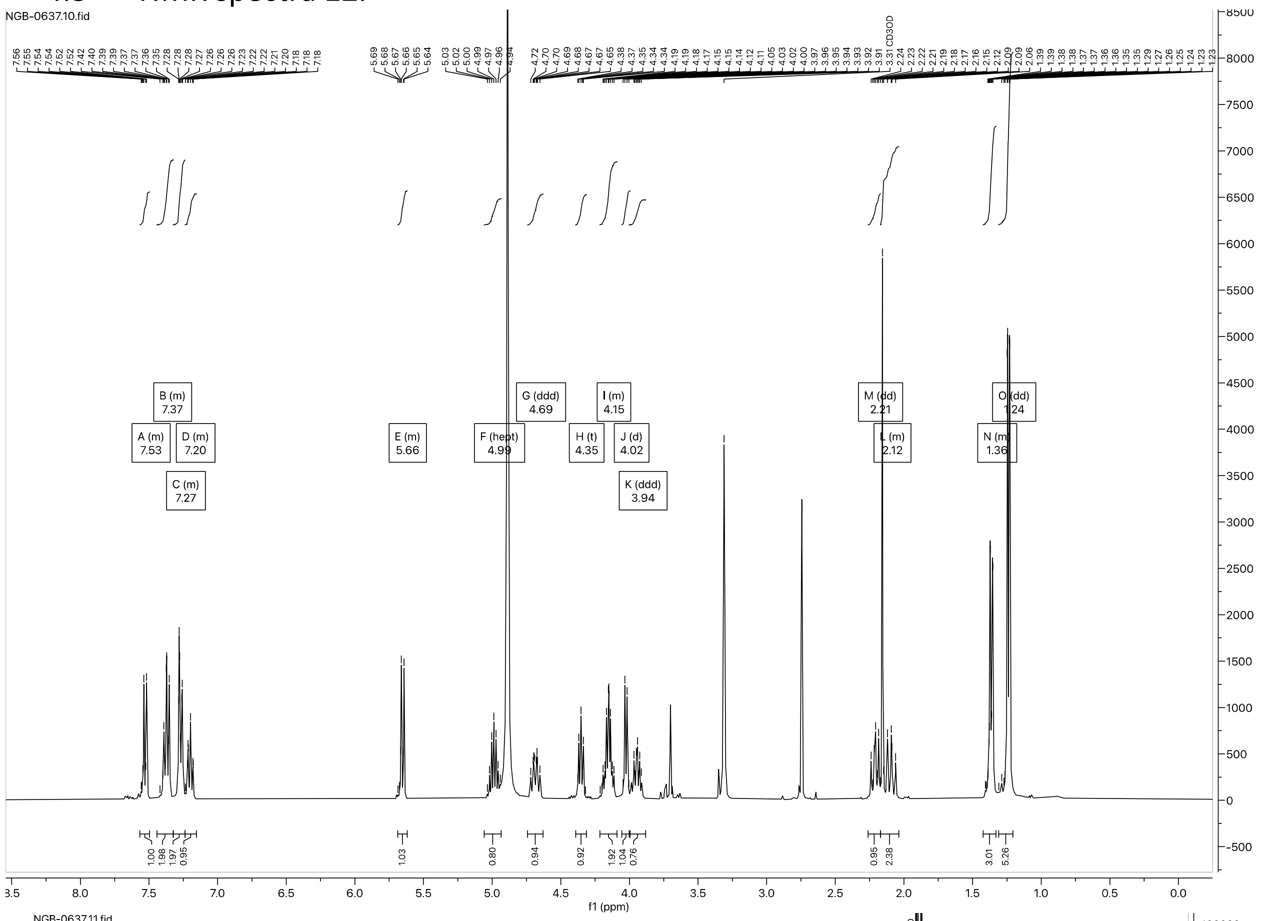

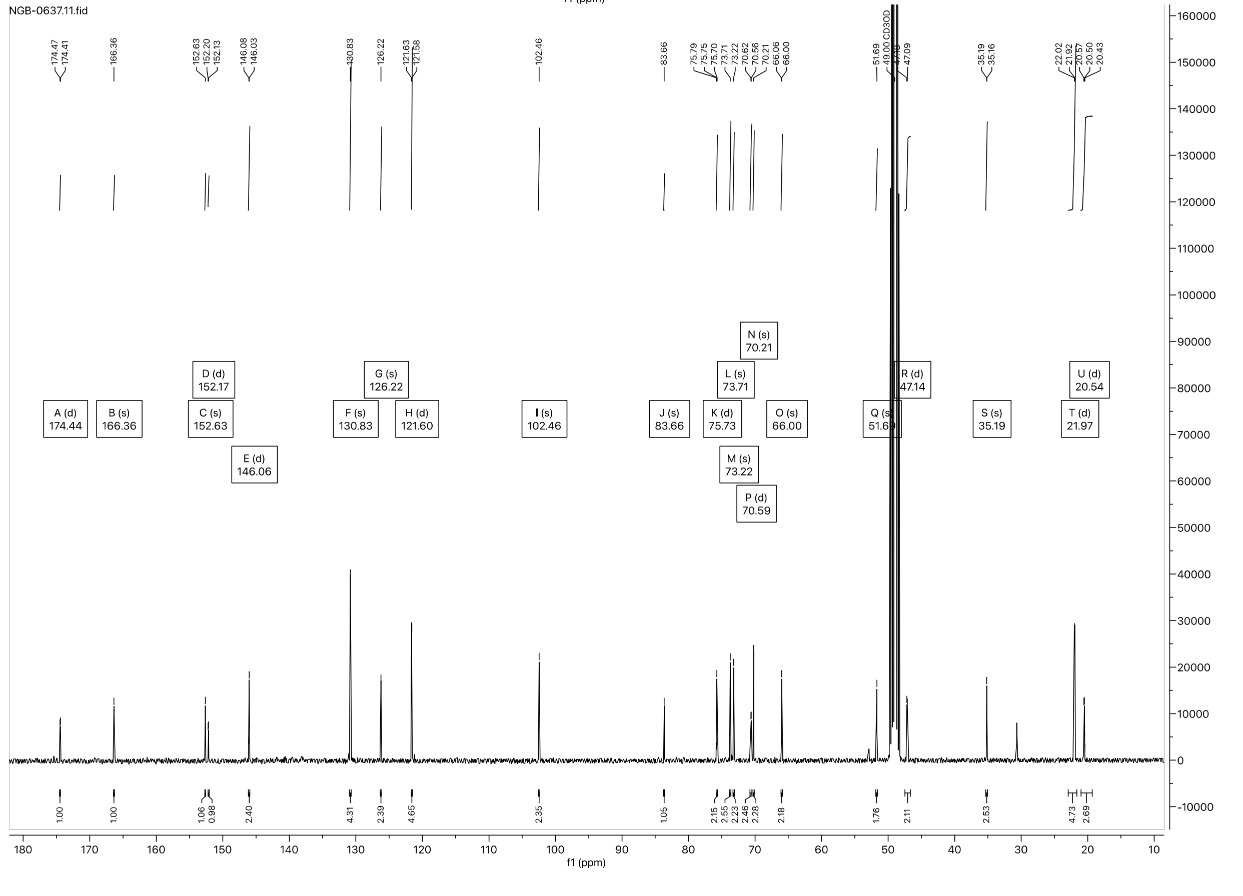

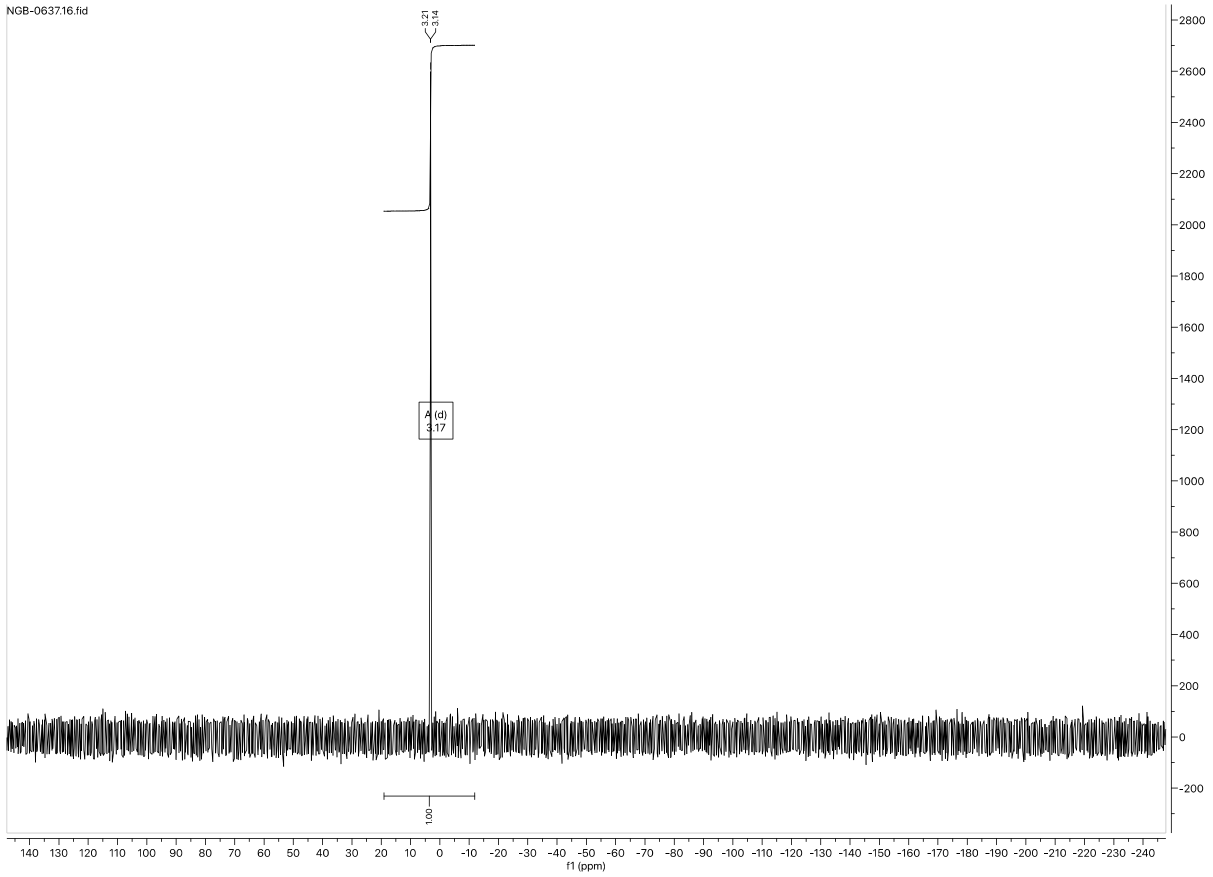

4.4- NMR spectra **13**.

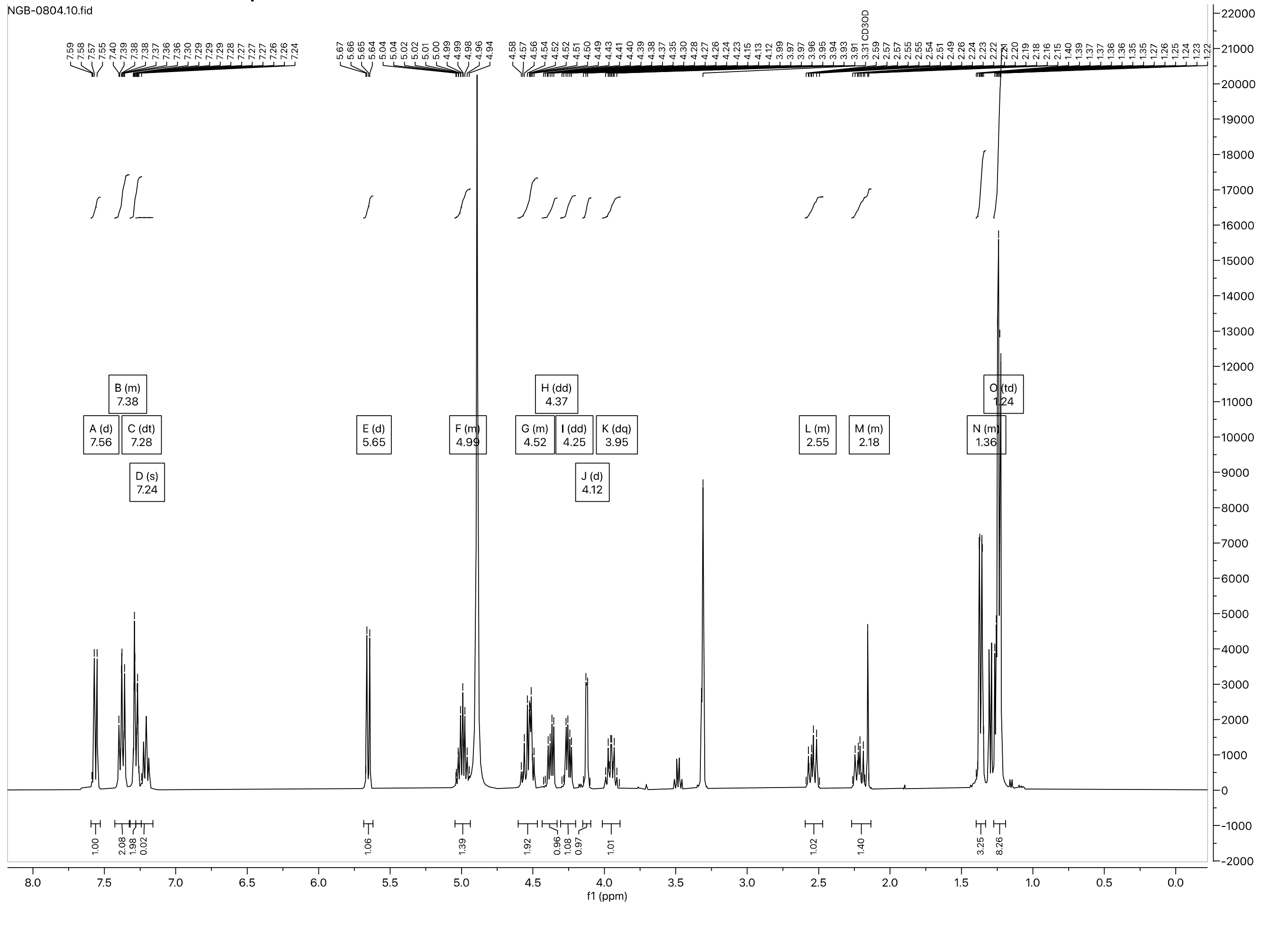

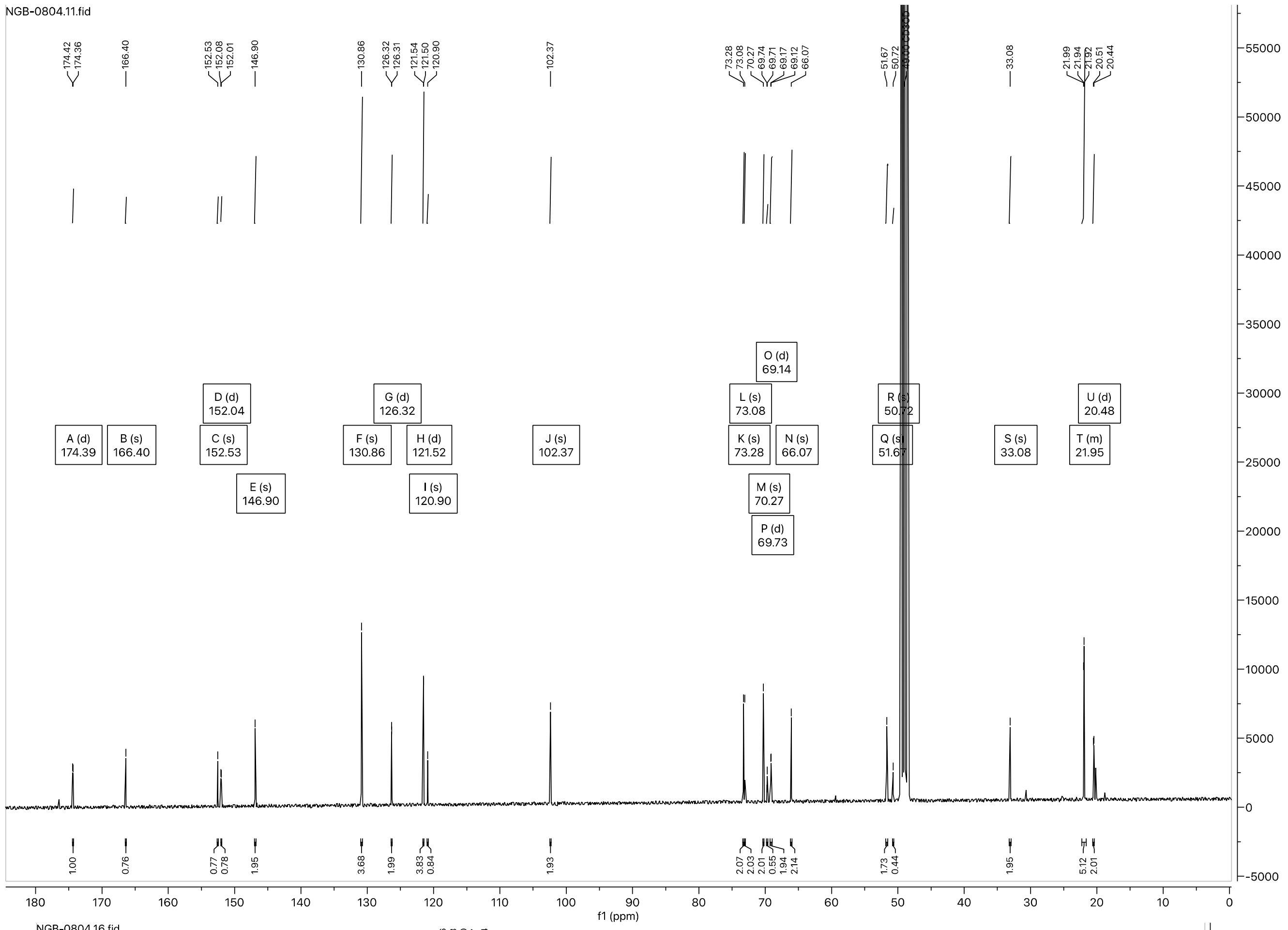
NGB-0804.11.fid
174.42
174.36
166.40
152.53
152.08
152.01
146.90
130.86
126.32
126.31
121.54
121.50
120.90
102.37
73.28
73.08
70.27
69.74
69.71
69.17
69.12
66.07
51.67
50.72
33.08
21.99
21.94
21.92
20.51
20.44
A (d) 174.39
B (s) 166.40
C (s) 152.53
D (d) 152.04
E (s) 146.90
F (s) 130.86
G (d) 126.32
H (d) 121.52
I (s) 120.90
J (s) 102.37
K (s) 73.28
L (s) 73.08
M (s) 70.27
N (s) 66.07
O (d) 69.14
P (d) 69.73
Q (s) 51.67
R (s) 50.72
S (s) 33.08
T (m) 21.95
U (d) 20.48
1.00
0.76
0.77
0.78
1.95
3.68
1.99
3.83
0.84
1.93
2.07
2.03
2.01
0.55
1.94
2.14
1.73
0.44
1.95
5.12
2.01
f1 (ppm)

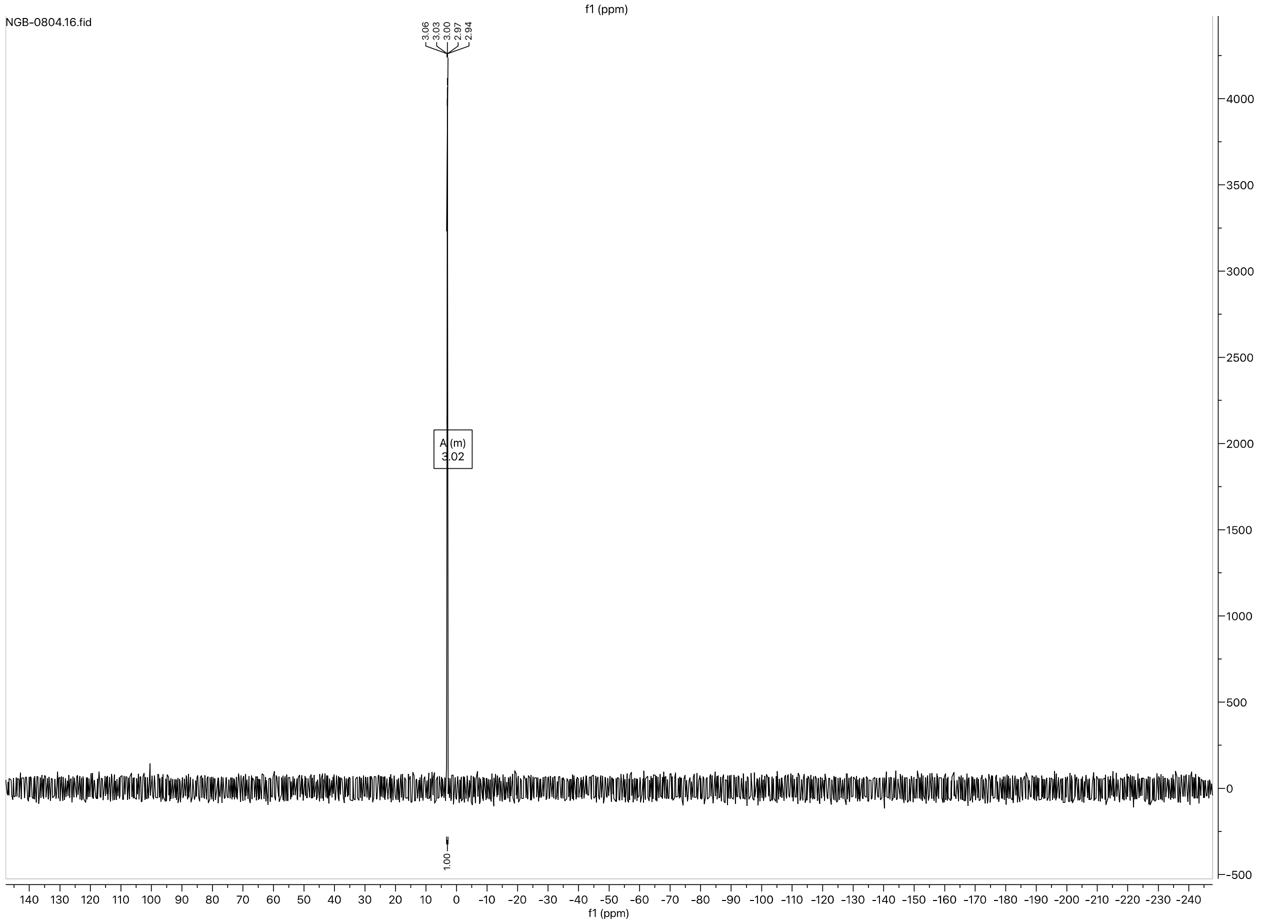
NGB-0804.16.fid
3.06
3.03
3.00
2.97
2.94
A (m) 3.02
1.00
f1 (ppm)

4.5- NMR spectra **14**.

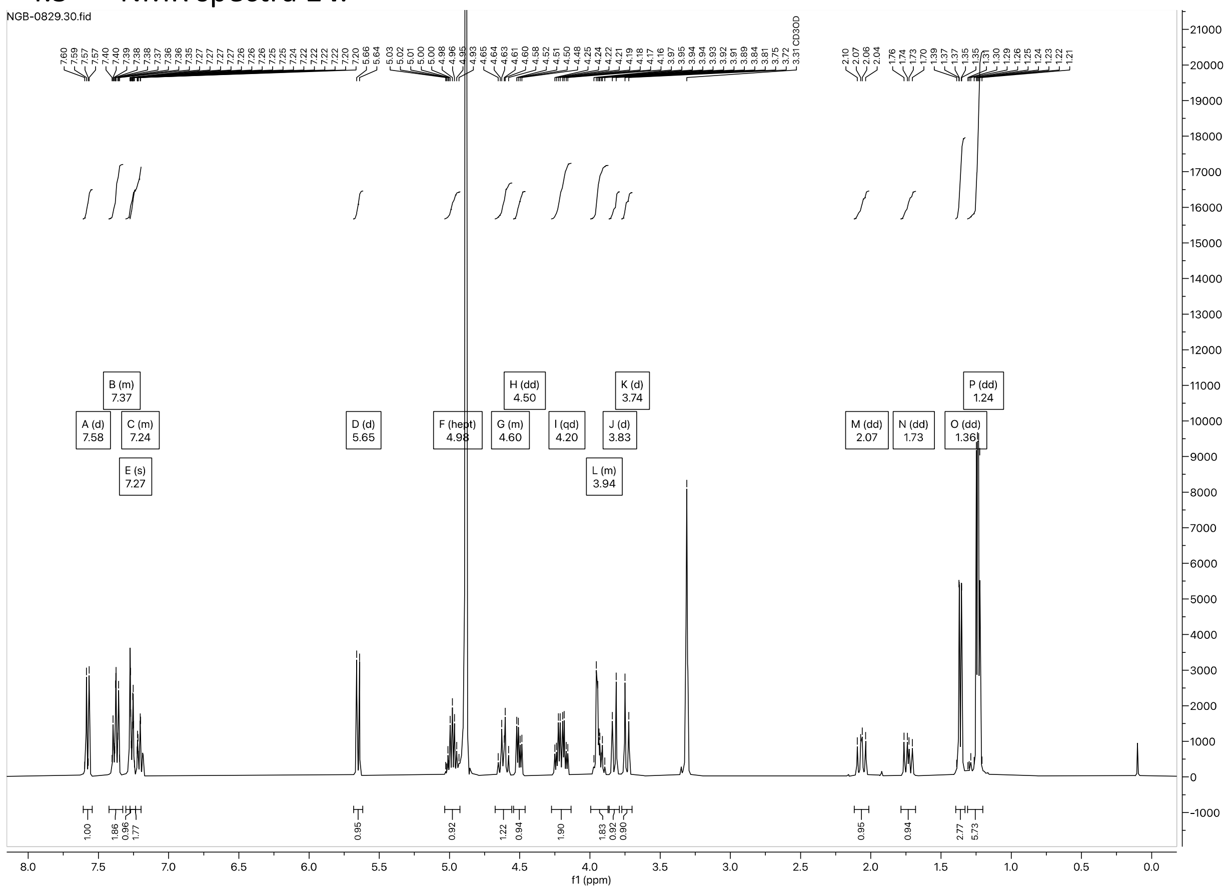

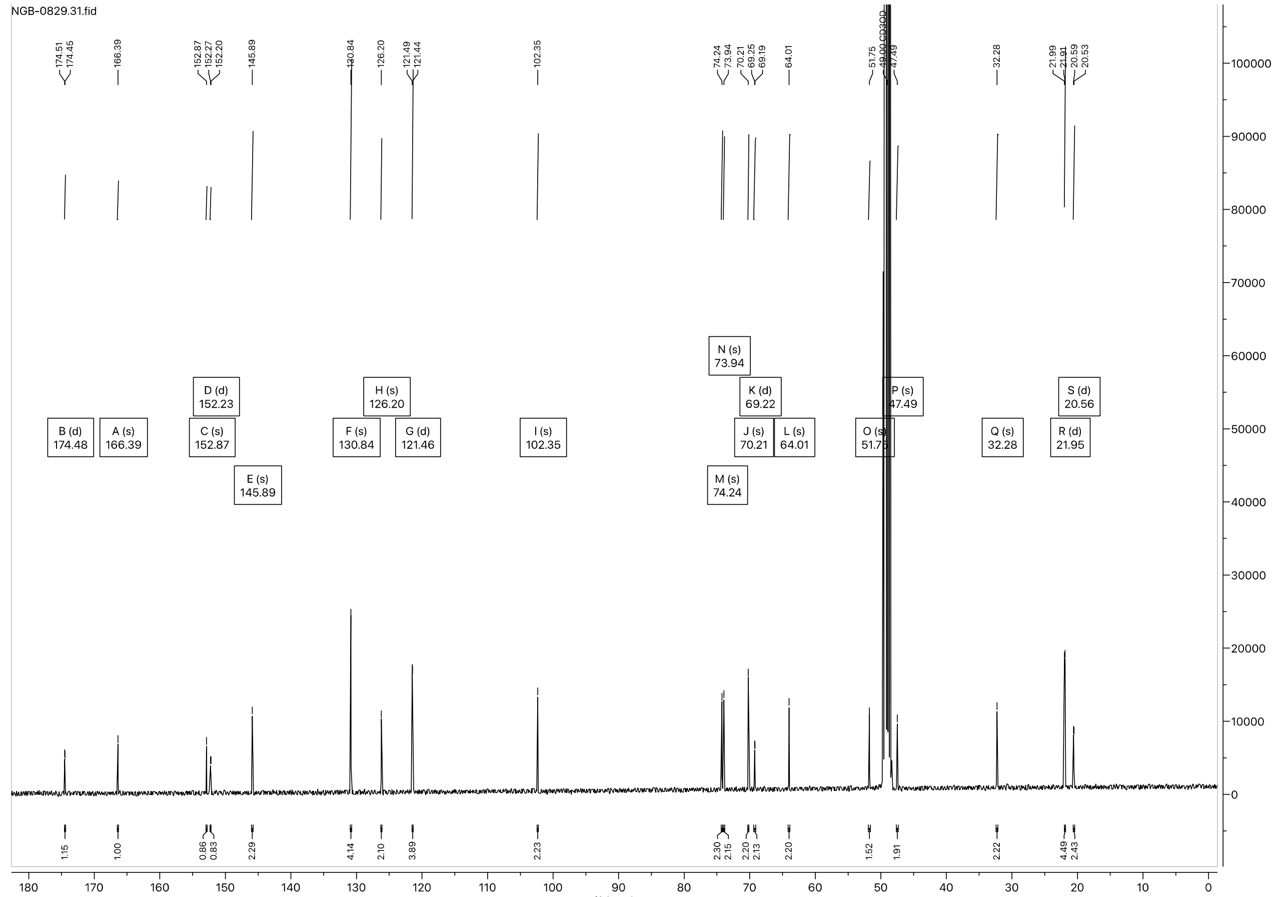

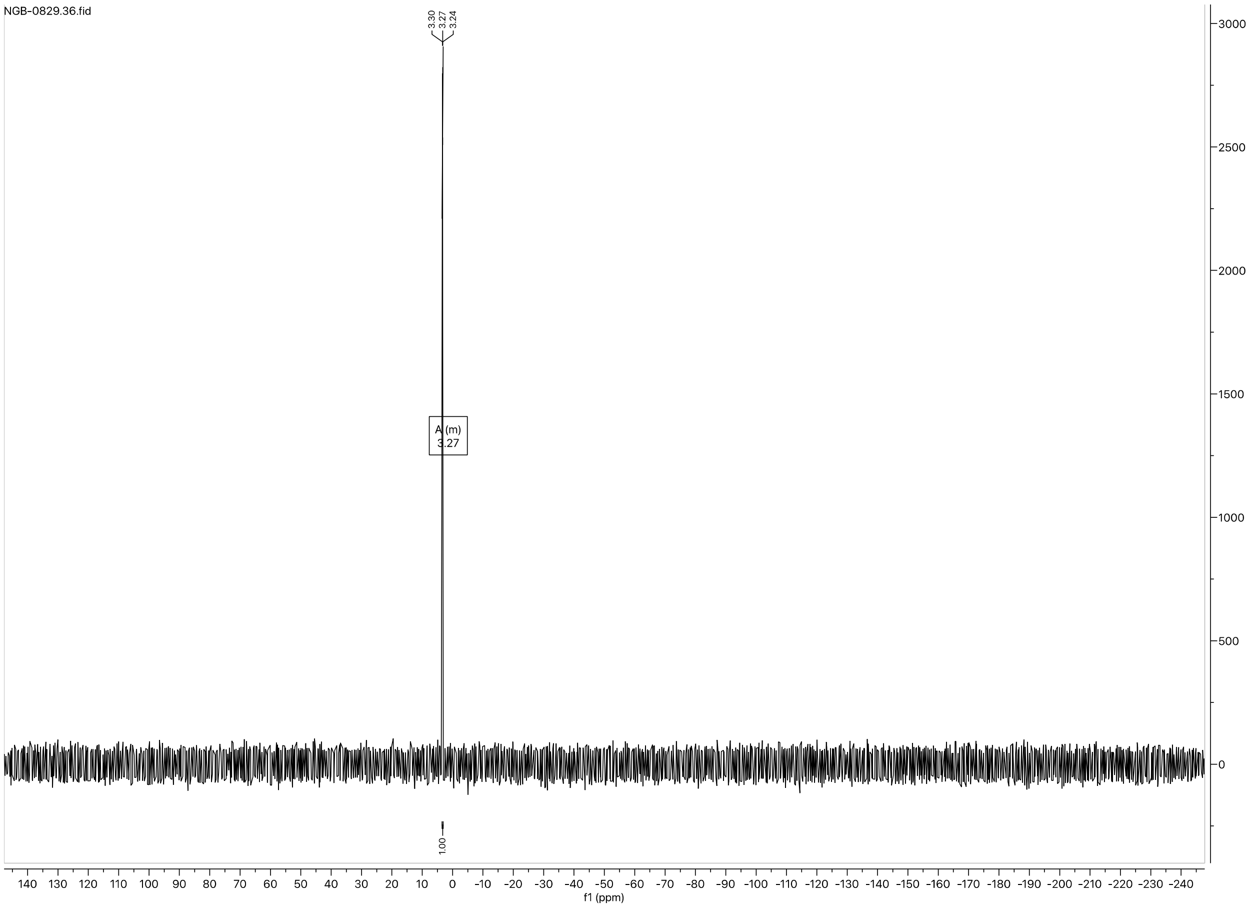

1. Liu, J.; Du, J.; Wang, P.; Nagarathnam, D.; Espiritu, C.L.; Bao, H.; Murakami, E.; Furman, P.A.; Sofia, M.J. A 2′- deoxy-2′-fluoro-2′-C-methyl uridine cyclopentyl carbocyclic analog and its phosphoramidate prodrug as inhibitors of HCV NS5B polymerase. *Nucleosides, Nucleotides Nucleic Acids*. **2012**, *31*, 277-285.
2. Sato, T.; Tsuzuki, T.; Takano, S.; Kohtaro, Fukuda, K.H.; Arisawa, M.; Shuto, S. Construction of a chiral quaternary carbon center by a radical cyclization/ring-enlargement reaction: synthesis of 4α-azidoethyl carbocyclic ribose, a key unit for the synthesis of cyclic ADP-ribose derivatives of biological importance. *Tetrahedron*, **2015**, *71,* 5407-5413.
3. Akabane-Nakata, M.; Chickering, T.; Harp, J. M.; Schlegel, M. K.; Matsuda, S.; Egli, M.; Manoharan, M. *Org. Lett.* **2022**, *24*, 2, 525–530.
